# Upregulated Expression of IL2RB Causes Disorder of Immune Microenvironment in Patients with Kawasaki Disease

**DOI:** 10.1155/2022/2114699

**Published:** 2022-07-25

**Authors:** Yunfei Liao, Ben Ke, Xiaoyan Long, Jianjun Xu, Yongbing Wu

**Affiliations:** ^1^Department of Cardiovascular Surgery, The Second Affiliated Hospital of Nanchang University, Nanchang, China; ^2^Department of Nephrology, The Second Affiliated Hospital of Nanchang University, Nanchang, China; ^3^East China Digital Medical Engineering Research Institute, Shangrao, China

## Abstract

**Aims:**

The clinical diagnosis of Kawasaki disease (KD) is not easy because of many atypical manifestations. This study is aimed at finding potential diagnostic markers and therapeutic targets for KD and analysing their correlation with immune cell infiltrations.

**Methods:**

First, we downloaded the KD dataset from the Gene Expression Omnibus (GEO) database and used R software to identify differentially expressed genes (DEGs) and perform functional correlation analysis. Then, CIBERSORT algorithm was used to evaluate immune cell infiltrations in samples. Coexpression analysis between DEGs and infiltrating immune cells was performed to screen the main infiltrating immune cells. Subsequently, the least absolute shrinkage and selection operator (LASSO) logistic regression analysis was used to screen the core genes related to KD. Finally, correlation analysis between the core genes and the main infiltrating immune cells was performed.

**Results:**

327 DEGs were screened out in this study. Among them, 72 shared genes were the category of genes most likely to be disease-causing for they did not change before and after treatment. After analysis, it was found that expression level of IL2RB in KD tissues was significantly upregulated, the number of resting CD4+ memory T cells was decreased, and the decrease was significantly negatively correlated with the upregulated expression of IL2RB. Therefore, it was speculated that the upregulated expression of IL2RB disrupted Th1/Th2 cell differentiation balance, which led to a decrease of resting CD4+ memory T cells and finally caused disorder of immune microenvironment in patients with KD.

**Conclusions:**

Upregulated expression of IL2RB leads to disorder of immune microenvironment in patients with KD and eventually causes the occurrence and development of KD. Therefore, IL2RB may serve as a diagnostic marker and potential therapeutic target for KD.

## 1. Introduction

Kawasaki disease (KD) is a rare systemic inflammatory disease that predominantly affects children less than 5 years old [1]. The main pathological feature of KD is an acute febrile rash accompanied by systemic vasculitis [2]. The leading theory for KD pathogenesis is that unknown stimuli trigger an immune-mediated inflammatory cascade in genetically susceptible children [3]. The most serious complication of KD is the occurrence of coronary artery abnormalities, and patients with atypical KD are also at risk [3]. KD has become the most common cause of acquired heart disease among children, in whom coronary artery abnormalities can cause myocardial ischemia, infarction, and even death [4].

In recent years, an increasing number of studies have shown that immune-mediated systemic vasculitis plays an important role in the occurrence and development of KD-related vascular complications [5]. The increases in the number of peripheral blood neutrophils, monocytes, and activated T cells indicate that the innate immune response is excessive in the acute phase of KD [6]. The recurrence of KD is usually observed within the first 12 months after the first attack [7], which indicates that the immune response of KD may lack immune memory and supports the hypothesis that the innate immune system participated in the pathogenesis of the disease [8]. The identification of pathogen-associated molecular patterns (PAMPs) in the serum of patients with KD provides new insights into the mechanism of vascular inflammation [8]. The elevations of S100 and HMGB1 protein levels in KD patients suggest the activation of endothelial cells and neutrophils by PAMPs [9].

Abnormalities in the adaptive immune response were also noted in patients with KD [10]. Microarray studies have shown that B and T cell receptor signaling pathways are downregulated [11]. Recent literatures have also documented increases in the levels of proinflammatory cytokines and chemokines during the acute phase of KD [12]. In addition, autopsy studies have shown that lymphocytes and macrophages had infiltrated the coronary artery wall 10 days before the onset of the disease [13]. Cytokines secreted by activated macrophages, T lymphocytes, and myofibroblasts can cause damage to the elastic layer and collagen fibres, leading to the occurrence of coronary aneurysms (CAAs) [14].

A large number of literatures have proven the correlation between KD and immunity, and some literatures have pointed out that immune dysfunction was the culprit [10]. However, which genes' mutation causes immune dysfunction, and which immune cells' dysfunction causes the disease? How does immune dysfunction lead to systemic vasculitis? Are there valid diagnostic markers to reduce clinical misdiagnosis? These issues remain to be elucidated. We consulted many literatures to seek the answers. A surprising discovery was made in the analysis upon the Gene Expression Omnibus (GEO) dataset GSE64486 [15]. Firstly, the microarray dataset of KD from the GEO database was downloaded and differentially expressed gene (DEG) analysis and functional annotations were performed. Then, CIBERSORT was used to evaluate the immune cell infiltrations in 22 subtypes of immune cells. In addition, the coexpression analysis between DEGs and infiltrating immune cells was conducted to screen the most important immune cells and better understand the molecular immune mechanism during the development of KD. Subsequently, the least absolute shrinkage and selection operator (LASSO) logistic regression analysis was used to further screen core genes related to KD. Finally, we identified diagnostic markers by correlation analysis between these core genes and the main infiltrating immune cells. Surprisingly, it was found that the decrease in the number of resting CD4+ memory T cells was significantly negatively correlated with upregulated expression of IL2RB in patients with KD. Therefore, it was speculated that the mutation of IL2RB caused its upregulation and disrupted Th1/Th2 cell differentiation balance, which led to a decrease in the number of resting CD4+ memory T cells and eventually caused disorder of immune microenvironment and KD occurrence. Therefore, IL2RB may serve as a diagnostic marker and potential therapeutic target for KD.

## 2. Materials and Methods

### 2.1. Data Introduction and Preprocessing

**Figure 1:**
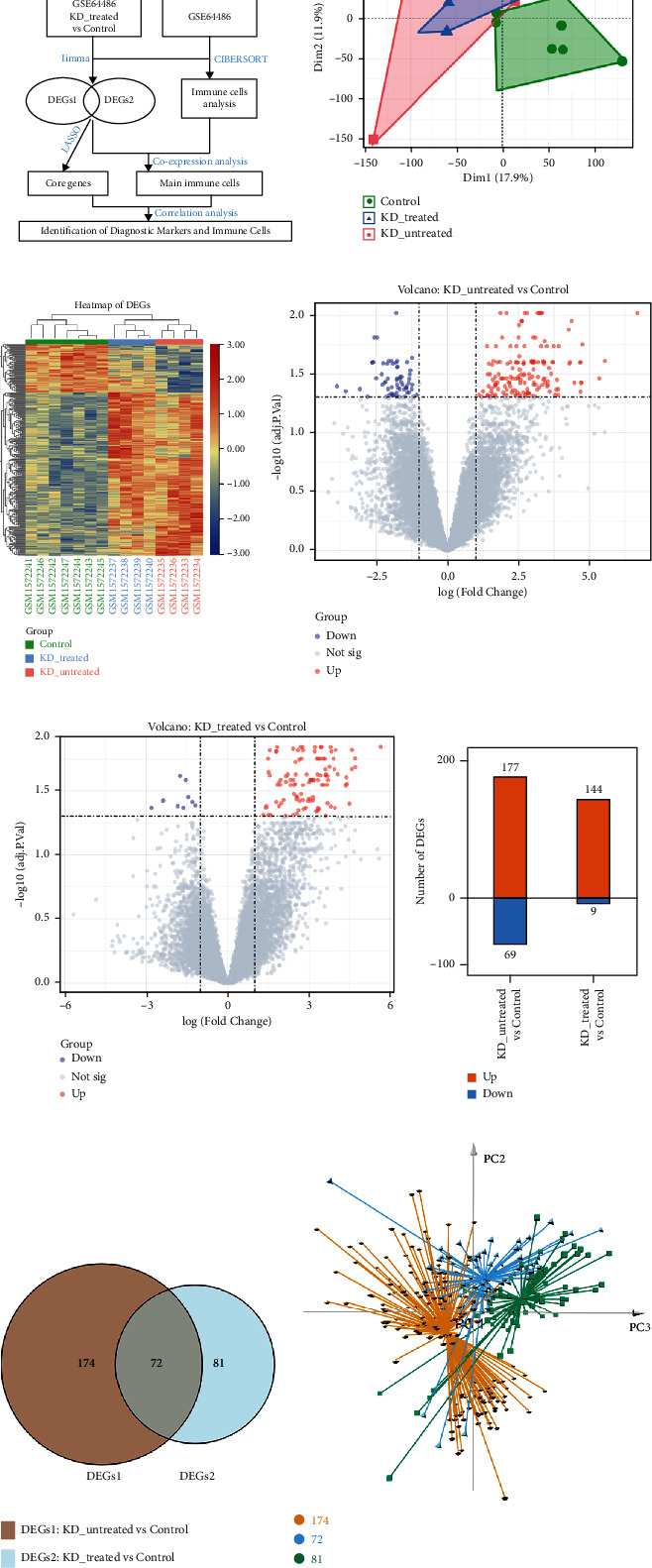
Data quality assessment, differentially expressed gene (DEG) extraction and subgroup analysis. (a) Workflow of this study. (b) Principal component analysis (PCA) of Kawasaki disease (KD) case group vs. control samples based on normalized gene expression level in the GSE64486 dataset. (c) The cluster heat map of the whole DEGs (including DEGs1 and DEGs2). DEGs1 were extracted from the comparison between the untreated group and the control group, and DEGs2 were extracted from the comparison between the treated group and the control group. (d, e) The volcano maps of DEGs1 and DEGs2. (f) The number of DEGs. The number of upregulated and downregulated DEGs was showed in bar plot. (g) The Venn diagram showing the overlapping genes between DEGs1 and DEGs2. (h) Three-dimensional PCA of these three subtypes of genes.

The workflow of this study is shown in Figure 1(a). “Kawasaki disease (KD)” was used as a keyword to retrieve and select the dataset that met the requirements. The Gene Expression Omnibus (GEO) database (https://www.ncbi.nlm.nih.gov/geo/) was searched. The “GEOquery” package of R software (version 4.0.4, http://r-project.org/) [16] was used to download the KD dataset GSE64486. The data from childhood coronary artery samples in the form of expression chip data were collected. The expression profiles were converted and standardized by log_2_ to obtain a series matrix file. The microarray data of GSE64486 were based on the GPL11154 platform (Illumina HiSeq 2000 (Homo sapiens)). High-throughput RNA sequencing (HTS) was performed on KD (*n* = 8) and childhood control (*n* = 7) coronary artery tissues [15]. Coronary artery tissues of KD children were obtained from biopsy or transplantation; control coronary artery tissues were obtained from children who need biopsy or transplantation due to other diseases. The clinical data of these children mentioned in this study were shown in the Supplementary Table 1. Among the 8 patients with KD, 4 were from children who had been treated, and the other 4 had not been treated. Coronary arteries from KD children were individually embedded at autopsy/transplant, while control epicardial coronary arteries were microdissected from myocardial tissue blocks and reembedded prior to sectioning [15].

### 2.2. Principal Component Analysis (PCA) and Differentially Expressed Gene (DEG) Screening

We used the “affy” package [17] to read the raw data of the GSE64886 dataset and utilized the Robust Multiarray Average (RMA) algorithm (https://www.bioconductor.org/) to correct background and normalize data [18]. PCA is a multiple regression analysis and was used to assess the quality of the data [19]. The “factoextra” package in R was used for data processing, analysis, and mapping. The effect of data correction was demonstrated using a two-dimensional PCA cluster plot. The differential expressions between the case and control groups were observed using DEGs as variables. DEGs were screened by the “limma” package [20]; heat map and volcano maps of DEGs were drawn using the “ggplot2” package to visualize the differential expression. DEGs with *P* < 0.05 and |log2 *FC*| > 1 were considered statistically significant.

### 2.3. Gene Functional Enrichment Analysis

“Metascape” (https://metascape.org/) was used to perform Gene Ontology (GO) enrichment analyses on DEGs. Protein-protein interaction (PPI) network and Molecular Complex Detection (MCODE) algorithm analyses were performed using “Cytoscape” software (version 3.7.2, https://cytoscape.org/). The clueGO module in “Cytoscape” software was used to perform the Kyoto Encyclopedia of Genes and Genomes (KEGG) enrichment analysis for DEGs.

### 2.4. Evaluation of Immune Cell Infiltrations

As required by code, the gene expression matrix data was uploaded to CIBERSORT to obtain the immune score matrix, with filtering out the samples with *P* < 0.05. Then, “ggplot2” package was used to draw a two-dimensional PCA clustering map to visualize the result on immune cell score matrix data. Then, we used the “corrplot” package [21] to draw a correlation heat map to visualize the correlations of 22 subtypes of infiltrating immune cells and utilized the “ggplot2” package to draw boxplot diagrams to show the differences of immune cell infiltrations between case and control groups.

### 2.5. Coexpression Analysis of DEGs and Immune Cell Populations

Coexpression analysis of DEGs and immune cell populations in the coronary artery tissues of KD and control children was carried out. We calculated Pearson correlation coefficients between DEGs and immune cell infiltrations in the dataset. An absolute value of Pearson's coefficient greater than 0.6 and *P* < 0.05 were used as enrichment criterions. Finally, combined with the differentially expressed analysis of immune cell components, the immune cell components most likely to be involved in the disease were screened out for subsequent analysis.

### 2.6. Screening and Verification of Diagnostic Markers

We used the least absolute shrinkage and selection operator (LASSO) logistic regression [22] to perform feature selection to screen the core genes for KD. The LASSO algorithm was applied by the “glmnet” package [23]. Finally, the correlation analysis between the core genes and the main immune cell components was further performed by the “corrplot” package, and the “ggplot2” package was used to draw a chord diagram for visualizing the results. A two-sided *P* < 0.05 was considered to be statistically significant.

## 3. Results

### 3.1. Principal Component Analysis (PCA) and Identification of Differentially Expressed Genes (DEGs)

The gene expression matrix of GSE64486 dataset was normalized and processed, and it is presented in a two-dimensional PCA cluster plot (Figure 1(b)), which showed that the three groups of samples were subclustered more obviously after normalization, indicating that the data were reliable. After data preprocessing, a total of 246 DEGs were defined as DEGs1, which were extracted from the comparison between the untreated case group and the control group (Supplementary Table 2); another 153 DEGs were defined as DEGs2, which were extracted from the comparison between the treated case group and the control group (Supplementary Table 3). All the DEGs were visualized by the heat map (Figure 1(c)) and volcano maps (Figures 1(d) and 1(e)).

177 genes were upregulated and 69 genes were downregulated in DEGs1, while 144 genes were upregulated and 9 genes were downregulated in DEGs2 (Figure 1(f)). The top 15 remarkably expressed genes between the case groups and control group are shown in Tables 1 and 2, respectively. The overlapping Venn diagram of DEGs1 and DEGs2 showed that 72 genes' expression levels did not change after treatment, 174 genes' expression differences disappeared after treatment, and 81 genes showed their expression differences only after treatment (Figure 1(g)). The three-dimensional PCA result showed that these three types of genes were obviously subclustered after normalization (Figure 1(h)). Among these three subgroups of genes, 72 genes' expression levels did not change after treatment, which were the category of genes most likely to be disease-causing. Therefore, these genes were selected for further gene functional annotations.

### 3.2. Functional Annotations for DEGs

**Figure 2:**
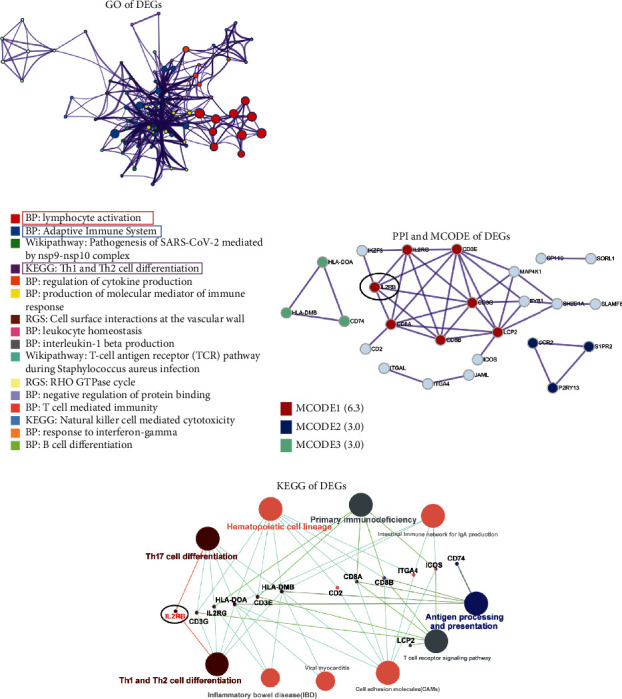
Functional annotations of differentially expressed genes (DEGs). (a) Top 16 most enriched Gene Ontology (GO) terms of DEGs. (b) Major genes and important functional modules were shown in protein-protein interaction (PPI) network. (c) Kyoto Encyclopedia of Genes and Genomes (KEGG) analysis revealed the KEGG signaling pathways and key genes.

Gene Ontology (GO) enrichment analysis results showed that DEGs were mainly related to lymphocyte activation, adaptive immune system, pathogenesis of SARS-CoV-2 mediated by the nsp9-nsp10 complex, and Th1 and Th2 cell differentiation (Figure 2(a)). The results displayed in Figure 2(b) showed the network of protein-protein interactions (PPIs) and the main Molecular Complex Detection (MCODE). The genes participating in the most important MCODE (with a score of 6.3) are IL2RB, IL2RG, CD3E, CD3G, CD8A, CD8B, and LCP2. IL2RB also participates in the signaling transduction process of two main signaling pathways associated with KD—Th1/Th2 cell differentiation and Th17 cell differentiation—and acts as a “bridge” to communicate these two signaling transduction pathways (Figure 2(c)). Most of the genes mentioned above are related to immunity, indicating that immune response plays an important role in KD.

### 3.3. Analysis of Immune Cell Populations

**Figure 3:**
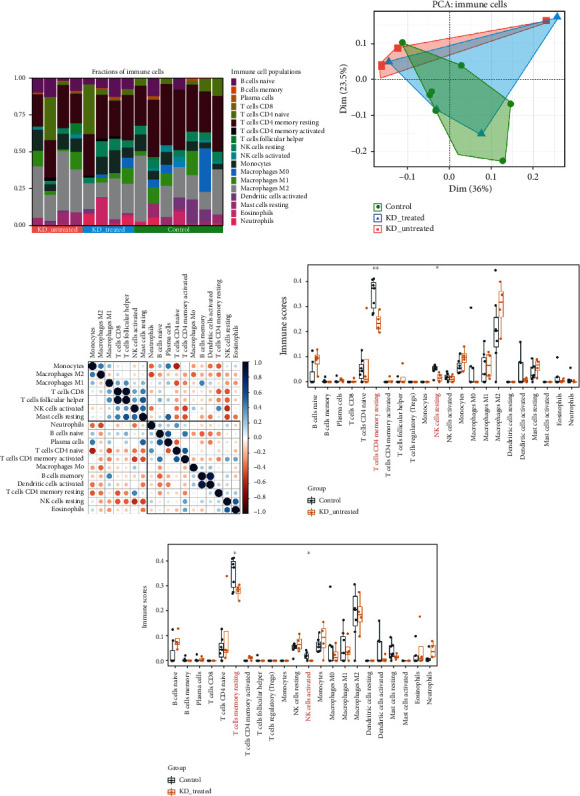
Immune cell infiltration analysis of Kawasaki disease (KD) vs. control group tissues. (a) Fractions of cell type estimated by CIBERSORT algorithm in each sample. (b) Principal component analysis (PCA) of immune score data based on different subgroups. (c) Correlation heat map of the 22 subtypes of immune cells. (d, e) The boxplot showed the difference in the number of infiltrating immune cells between case and control groups. Immune cells with statistically significant differences in the number of infiltrations were highlighted in red. ∗∗ represents *P* < 0.01, and ∗ represents *P* < 0.05.

We analysed the differences in the number of immune cell infiltrations in childhood coronary artery tissues of KD patients vs. controls. The fractions of the various cell types were estimated by CIBERSORT algorithm (Figure 3(a); Supplementary Table 4), and the immune score data quality were assessed by two-dimensional PCA. There was an obvious difference of immune cell infiltrations between KD and control tissues as shown in PCA cluster analysis plot (Figure 3(b)). A correlation heat map of the 22 subtypes of immune cells revealed that naive CD4+ T cells had a significantly positive correlation with monocytes, and activated natural killer (NK) cells also had a positive correlation with resting NK cells. Naive B cells and plasma cells, CD8+ T cells and follicular helper T cells, plasma cells and resting mast cells, memory B cells and activated dendritic cells, naive CD4+ T cells and activated CD4+ memory T cells, and follicular helper T cells and resting mast cells had significantly negative correlations (Figure 3(c)). Box plots of differences in the number of immune cell infiltrations showed that fewer resting CD4+ memory T cells and NK cells infiltrated in the untreated and treated KD tissues than that in control tissues (Figures 3(d) and 3(e); *P* < 0.05).

### 3.4. Coexpression Analysis of DEGs and Immune Cell Populations

**Figure 4:**
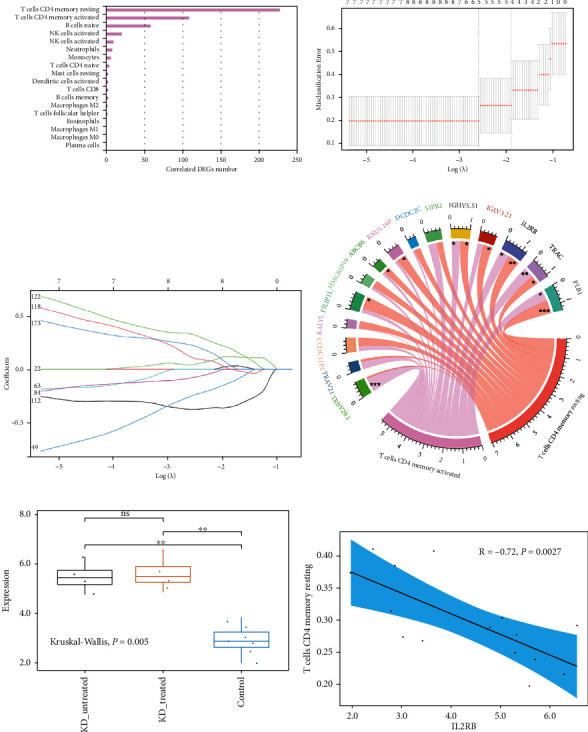
Screening and verification of diagnostic markers for patients with Kawasaki disease (KD). (a) Coexpression analysis of DEGs and immune cell populations. (b) CV (coefficient of variation) statistical graph showed the optimal lambda (*λ*, dotted line on the left). (c) Regression model built by the optimal *λ* screened 15 core genes related to KD. (d) The chord diagram showed the correlation and their statistical significances between these 15 core genes and the two most important immune cell components. (e) The box plot showed the differences of IL2RB expression between the case groups and the control group. Kruskal–Wallis tests was used to analyse the significance. (f) Correlation analysis between IL2RB expression and the number of resting CD4+ memory T cells. ∗∗∗ represents *P* < 0.001, ∗∗ represents *P* < 0.01, ∗ represents *P* < 0.05, and ns represents *P* > 0.05.

Through the above analysis, it was found that there were significant differences in the infiltrating numbers of resting CD4+ memory T cells and NK cells between the case groups and the control group. A coexpression analysis of DEGs and immune cell populations was conducted to further clarify the immune cell subtypes that play a major role in the pathogenesis of KD and their correlation with DEGs.  Figure 4(a) and Supplementary Table 5 show the immune cell populations and the coexpressed DEGs. The results showed that CD4+ memory T cells were enriched with the largest number of DEGs, indicating that CD4+ memory T cells may be protagonists in the occurrence of KD and that the change in the number of CD4+ memory T cells may reflect the pathogenesis of KD. Therefore, we selected CD4+ memory T cells for further analysis.

### 3.5. Screening and Verification of Diagnostic Markers

We used the LASSO logistic regression model to identify 15 core genes from DEGs as diagnostic markers for KD (Figures 4(b) and 4(c)); then, the correlations between the 15 core genes and CD4+ memory T cells were further analysed. The result of correlation analysis showed that IL2RB, PLB1, TRAC, and IGHV5-51 were both significantly correlated with resting CD4+ memory T cells and activated CD4+ memory T cells (Figure 4(d); Supplementary Table 6). Among these genes, IL2RB attracted our attention. IL2RB is one of the core members in the most important functional modules corresponding to DEGs and is also a core member of the main DEG-enriched KEGG signaling pathways. IL2RB expression levels in the treated and untreated case groups of KD were both significantly higher than those in the control group (Figure 4(e); *P* = 0.005). IL2RB's high expression level was significantly negatively correlated with the decrease in the number of resting CD4+ memory T cells (Figure 4(f), *R* = −0.72, *P* = 0.0027).

## 4. Discussion

Kawasaki disease (KD) is a systemic vascular inflammatory disease that was first reported by Dr. Tomisaku Kawasaki in 1967 [24]. It was once called mucocutaneous lymph node syndrome (MCLS) [25]. The epidemiology of KD varies greatly by geographic location and seasonality [3]. The highest incidence rates were observed in children of Japanese ancestry, followed by those of South Korean ancestry, also among children under five years with a male predominance [3]. A significant ethnic variation was observed, with the highest rates among Asian/Pacific Islanders [3].

Systemic vasculitis is the disease's basic pathological process, which mainly impairs the large and medium blood vessels [26]. Coronary artery diseases are serious complications that can lead to ischemia, myocardial infarction, and sudden death, among which coronary artery aneurysms (CAAs) and coronary artery stenosis are the most serious [27]. KD has replaced rheumatic fever as the primary cause of childhood-acquired cardiovascular diseases [28]. This disease has attracted people's attention because of its serious complications, and its incidence in untreated children has reached 20–25% [28]. The exact cause of KD remains unknown, but it is thought to be contagious and subsequently activate the immune system in genetically susceptible individuals [27]. It is debatable whether KD is postinfectious hyperinflammation, autoinflammatory, or autoimmune disorders [29]. After years of research, KD is considered an immune-mediated vasculitis [30]. Studies have found that T cells are mainly involved in KD's immune pathogenesis, including increased T cell activation and cytokine production, the proinflammatory (Th cells) and anti-inflammatory cells (Treg cells) imbalance [31, 32]. In addition, matrix metalloproteinase 9 (MMP9) is activated by TNF-*α*, which leads to the destruction of elastin and aneurysms formation in the blood vessel wall [33], and increased NO concentration, which leads to the blood vessel wall's expansion and damage [34].

In recent years, many KD-related genes have been discovered, including inositol 1,4,5-triphosphate 3-kinase (ITPKC), caspase 3 (CASP3), B lymphocyte kinase (BLK), CD40, and human leukocyte antigen (HLA) [35–37]. The polymorphism of ITPKC may lead to increased T cell activation and therefore increase interleukin 2 (IL-2) release. This may cause long-term expression of T cells in KD's acute phase and cause vascular endothelial cell damage, subsequently increasing the risk of severe coronary artery disease in KD [35]. CASP3 gene mutations can inhibit T cell apoptosis and prolong the activation time of immune cells, thereby increasing the sensitivity of the immune system to KD [36].

Although KD is closely related to immunity, its exact causes and mechanisms remain unclear. The diagnosis of KD is mainly based on a constellation of clinical findings that appear in typical KD due to the lack of reliable confirmatory laboratory tests [38]. However, some children may have incomplete or atypical forms of KD, and diagnosis can often be difficult, especially in infants and young children [39]. Therefore, in order to clarify the possible pathogenesis of KD and seek possible diagnostic markers and potential therapeutic targets to assist clinical diagnosis and treatment, we consulted a large number of literatures and made a surprising discovery upon analysing the GSE64486 dataset using bioinformatics analysis. It was found that IL2RB expression level in KD tissues was significantly upregulated, the number of resting CD4+ memory T cells was decreased, and the decrease was significantly negatively correlated with the upregulated expression of IL2RB. Changes in IL2RB expression or its affinity to IL2 could affect Th1/Th2 cell differentiation balance [40]. Therefore, it was speculated that the mutation of IL2RB caused its upregulation and disrupted Th1/Th2 cell differentiation balance, which led to a decrease in the number of resting CD4+ memory T cells and caused the disorder of the immune microenvironment and KD occurrence. IL2RB may serve as a diagnostic marker and potential therapeutic target for KD.

Dataset GSE64486 from the Gene Expression Omnibus (GEO) database was downloaded and processed using R software. After normalization and deduplication, 327 differentially expressed genes (DEGs) were identified. Among them, there were 246 DEGs between the untreated case and control groups and 153 between the treated case and control groups. 72 shared DEGs were observed between the case (including the untreated and treated case groups) and control groups, which did not change after treatment and were also the category of genes most likely to be disease-causing. After preliminary processing, gene functional annotations were performed for these 72 shared DEGs. The result showed that these genes predominantly relate to lymphocyte activation, adaptive immune system, SARS-COV-2 pathogenesis mediated by the nsp9-nsp10 complex, and Th1 and Th2 cell differentiation. Subsequently, a protein-protein interaction (PPI) network was constructed, and their main functional modules were IL2RB, IL2RG, CD3E, CD3G, CD8A, CD8B, and LCP2. Previous studies have shown that these genes are closely correlated with immunity [41–45]. This indicates that the KD is closely correlated with immunity.

The results of gene functional annotations and Kyoto Encyclopedia of Genes and Genomes (KEGG) analysis showed that the DEGs participated in the occurrence and development of KD mainly through Th1 and Th2 cell differentiation signaling pathways. In this signaling pathway, naive CD4+ T cells differentiate into Th0 cells after antigen stimulation [46]. As precursors of Th1 and Th2 cells, Th0 cells secrete Th1- and Th2-like cytokines [46]. Differentiation of precursor Th0 cells into Th1 or Th2 cells requires repeated stimulation by antigens, and their differentiation is affected by factors such as the microenvironment and antigen-presenting cells (APCs) [47]. Cytokines play an important regulatory role in differentiation. The cytokines IL-4 and IL-13 mainly regulate Th2 cell differentiation, while IL2, IFN-*α*, IL-12, and IFN-*γ* can regulate Th1 cell differentiation [48]. In addition, the affinity of the major histocompatibility complex (MHC) antigen peptide T cell receptor (TCR) is also an important factor affecting the differentiation of Th1/Th2 cells [49]. The higher the affinity between the antigen peptide and MHC, the better the Th1 cell differentiation; and the lower the affinity, the better the Th2 cell differentiation. The higher the affinity between the antigen peptide-MHC complex and TCR, the better the Th1 cell differentiation, and vice versa [50]. The differentiation balance of Th1/Th2 cells has an important impact on the body's physiological functions [51]. Disruption of Th1/Th2 cell differentiation balance will lead to disease occurrence [51]. In the immune infiltration analysis of this study, resting CD4+ memory T cells and NK cells were significantly reduced among the 22 subtypes of immune cells in the case groups compared to the control group, which may be the main reason for immune dysfunction in KD. CD4+ T cells play an “auxiliary” role in the immune system [50]. In most cases, they cannot directly neutralize infection but guide and trigger the body's immune response to the infection, similar to “sentinels” of the immune system [52]. Therefore, CD4+ T cells are also called helper T (Th) cells. CD4+ T cells can be divided into naive CD4+, CD4+ effector, and CD4+ memory T cells according to their activation stage [53], and they can also be divided into Th1, Th2, Th17, Th22, Treg, and T follicular helper cell (Tfh) subtypes according to differences in secreted cytokines [53]. In this study, the Th1/Th2 cell differentiation imbalance led to a decrease in the number of resting CD4+ memory T cells, which eventually caused a disorder in the immune microenvironment of patients with KD.

Coexpression analysis of infiltrating immune cells and DEGs was performed to understand the correlation between decreased infiltration of CD4+ memory T cells and these DEGs. It was found that CD4+ memory T cells were enriched with the largest number of DEGs, indicating that these cells were the protagonists in KD's immune response. The least absolute shrinkage and selection operator (LASSO) logistic regression model for screening 15 core genes was used to identify the most critical DEGs. IGHV5-51, IL2RB, TRAC, and PLB1 were significantly related to CD4+ memory T cells when the correlations between these 15 core genes and CD4+ memory T cells were analysed. Among them, IL2RB attracted our attention because it showed its core position throughout the entire study analysis. Moreover, interleukin-2 (IL2) cytokines play an important role in Th1/Th2 cell differentiation as a stimulator of IL2RB [46]. In the case groups, the gene mutation significantly upregulated IL2RB expression. Interestingly, upregulated expression of IL2RB was significantly negatively correlated with a decrease in the number of resting CD4+ memory T cells. Therefore, upregulated expression of IL2RB is the culprit of imbalanced Th1/Th2 cell differentiation, which leads to a decrease in the number of resting CD4+ memory T cells.

**Figure 5:**
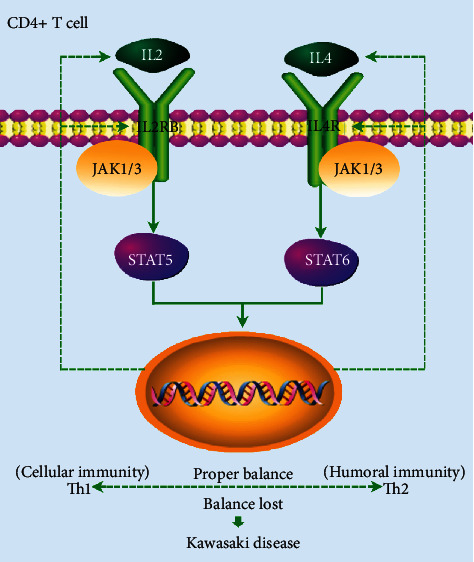
Schematic diagram of the mechanism.

IL2 is a glycoprotein with a molecular weight of 15 kDa. It is a type 1 quadruple alpha-helix bundle cytokine, mainly produced by CD4+ T cells after antigen stimulation, and exerts its effects in an autocrine and paracrine manner [54]. IL2 plays a critical role in regulating immune responses, primarily through the IL2/IL2R complex axis, and triggering a series of intracellular events, including the activation of JAK-STAT transcription signaling transduction [55]. IL2R comprises IL2R*α* (CD25), IL2R*β* (CD122), and IL2R*γ* (CD132) subunits [56, 57]. The low-affinity form is a monomer of the *α*-subunit in terms of its ability to bind to IL2 and does not participate in signaling transduction [58]. The medium-affinity form is composed of *α*/*β* subunit heterodimers, whereas the high-affinity form is composed of *α*/*β*/*γ* subunit heterotrimers [58]. Both the medium- and high-affinity forms of the receptor participated in receptor-mediated endocytosis and mitotic signaling transductions from IL2 [58]. Previous studies have shown that defects in the CD122 gene can cause inflammation in multiple organs in mice and humans [59–61]. Knockout mice of CD122 developed lethal autoimmune diseases due to a lack of Treg cells [62]. Importantly, IL2RB mainly combines with IL2 to play a key role in coronary heart disease (CHD) development [63], which indicates that it also plays a role in cardiovascular-related immune-inflammatory diseases. Based on the interesting phenomena found in this study, a possible mechanism by which upregulated expression of IL2RB cause KD's occurrence was concluded, as shown in Figure 5. After the upregulated expression of IL2RB, IL2/IL2R-JAK-STAT5 transcription signaling transduction is greatly activated and combines with the activation of IL4/IL4R-JAK-STAT6 transcription signaling transduction, resulting in massive IL2 cytokine secretion. IL2 cytokines induce target cells to increase the expression of IL2RB and cyclically stimulate CD4+ T cell differentiation. This process resulted in the conversion of resting CD4+ memory T cells into activated CD4+ memory T cells, which significantly reduced the number of resting CD4+ memory T cells and slightly increased the number of activated CD4+ memory T cells in KD tissues. Subsequently, activated CD4+ T cells further differentiate into other CD4+ T cells, such as Th1 and Th2 cells. This cyclic process eventually disrupts CD4+ T cell differentiation balance, which ultimately leads to a disorder of the immune microenvironment and KD occurrence. Therefore, combined with existing studies and the findings of this study, we believe that IL2RB may be responsible for the occurrence of KD and KD-related systemic vasculitis.

This study had some limitations, although the discovery was startling. The mechanism of KD occurrence after Th1/Th2 cell differentiation imbalance and how IL2RB causes systemic vasculitis has not been elucidated. Therefore, further studies are required to verify our conclusions.

## 5. Conclusions

In summary, KD's occurrence and development are significantly correlated with immune dysfunction—a decrease in the number of resting CD4+ memory T cells, which was caused mainly by the upregulated expression of IL2RB. The upregulated expression of IL2RB disrupts the Th1/Th2 cell differentiation balance, leading to the destruction of the immune microenvironment, eventually leading to KD's occurrence and development. Therefore, IL2RB may serve as a diagnostic marker and potential therapeutic target for KD, and disorders of the immune microenvironment may play an important role in its occurrence and development.

## Figures and Tables

**Table 1:** The top 15 upregulated and downregulated genes between the untreated group and the control group.

Upregulated genes			Downregulated genes		
Gene names	*P* value	logFC	Gene names	*P* value	logFC
IGHG1	7.96 × 10^−3^	6.68	LETM1	9.82 × 10^−3^	-1.80
CD74	7.96 × 10^−3^	3.24	NSFP1	1.34 × 10^−2^	-2.55
PLB1	7.96 × 10^−3^	1.85	HMGB1P24	1.58 × 10^−2^	-2.40
APOBEC3G	7.96 × 10^−3^	2.08	MIR1276	2.13 × 10^−2^	-1.77
IGHV3-11	7.96 × 10^−3^	3.22	HOOK2	2.13 × 10^−2^	-2.06
EPSTI1	7.96 × 10^−3^	2.80	SLC27A5	2.13 × 10^−2^	-2.60
OAS1	7.96 × 10^−3^	3.17	TOMM40	2.13 × 10^−2^	-1.97
TRAC	7.96 × 10^−3^	3.26	MLX	2.42 × 10^−2^	-1.24
MX1	7.96 × 10^−3^	3.31	GGN	2.42 × 10^−2^	-2.57
IGHV3-33	9.82 × 10^−3^	2.62	KCNG2	2.42 × 10^−2^	-2.02
IGKV1-5	9.82 × 10^−3^	4.27	MCAT	2.42 × 10^−2^	-1.42
IGLC7	9.82 × 10^−3^	2.71	TRMT61A	2.42 × 10^−2^	-1.81
IL2RB	9.82 × 10^−3^	2.60	C3orf27	2.42 × 10^−2^	-2.62
AOAH	9.82 × 10^−3^	2.62	RPS26P56	2.42 × 10^−2^	-1.77
IFIT2	1.07 × 10^−2^	2.52	IL20RB	2.51 × 10^−2^	-2.21

Note: logFC, log_2_ (fold change).

**Table 2:** The top 15 upregulated and 9 downregulated genes between the treated group and the control group.

Upregulated genes			Downregulated genes		
Gene names	*P* value	logFC	Gene names	*P* value	logFC
ADAMDEC1	1.01 × 10^−2^	5.59	MIR1276	2.25 × 10^−2^	-1.75
TRAC	1.01 × 10^−2^	3.74	S1PR2	2.63 × 10^−2^	-1.54
SORL1	1.01 × 10^−2^	3.46	PKDCC	3.75 × 10^−2^	-1.45
CD8A	1.01 × 10^−2^	3.41	RSPO4	3.86 × 10^−2^	-2.76
MNDA	1.01 × 10^−2^	3.22	CDC42EP4	3.90 × 10^−2^	-1.84
IL2RB	1.01 × 10^−2^	2.75	LETM1	4.25 × 10^−2^	-1.30
PLB1	1.01 × 10^−2^	1.82	OSGIN1	4.25 × 10^−2^	-1.62
TRBC2	1.38 × 10^−2^	3.96	SERPINA3	4.32 × 10^−2^	-3.32
SELL	1.44 × 10^−2^	4.71	CACFD1	4.56 × 10^−2^	-1.19
CD3G	1.44 × 10^−2^	4.57			
CD2	1.44 × 10^−2^	3.89			
CD8B	1.44 × 10^−2^	3.77			
IL2RG	1.44 × 10^−2^	3.53			
LOC101060038	1.44 × 10^−2^	3.53			
CCR2	1.44 × 10^−2^	3.35			

Note: logFC, log_2_ (fold change).

## Data Availability

The data used to support the findings of this study are available from the corresponding author upon request.

## Supplementary Materials

Supplementary Materials Supplementary Table 1: clinical data on children whose coronary artery tissues were tested in this study. Supplementary Table 2: the DEGs1 from the comparison between the untreated case group and the control group. Supplementary Table 3: the DEGs2 from the comparison between the treated case group and the control group. Supplementary Table 4: immune cell score matrix estimated by CIBERSORT algorithm. Supplementary Table 5: coexpression analysis of DEGs and immune cell populations. Supplementary Table 6: correlation analysis between the screened 15 core genes and CD4+ memory T cells.
