# Supplementary material for: Upregulated Expression of IL2RB Causes Disorder of Immune Microenvironment in Patients with Kawasaki Disease
Source: Biomed Res Int. 2022 Jul 25;2022:2114699. doi: 10.1155/2022/2114699 (PMC9343205; doi:10.1155/2022/2114699)
Supplement: Supplementary Materials — Supplementary Table 1: clinical data on children whose coronary artery tissues were tested in this study. Supplementary Table 2: the DEGs1 from the comparison between the untreated case group and the control group. Supplementary Table 3: the DEGs2 from the comparison between the treated case group and the control group. Supplementary Table 4: immune cell score matrix estimated by CIBERSORT algorithm. Supplementary Table 5: coexpression analysis of DEGs and immune cell populations. Supplementary Table 6: correlation analysis between the screened 15 core genes and CD4+ memory T cells. [file 2114699.f1.zip › Supplementary Table 1 (1).pdf]

S Table 1. Clinical data on children whose coronary artery tissues were tested in this study.

Case	Time since onset	Diagnosis	Therapy	Category	RNA tested by
KD1	2.5 weeks	KD	None	Untreated	HTS, PCR
KD2	4 weeks	KD	ASA, dipyridamole	Untreated	HTS
KD3	5 months	KD	None	Untreated	HTS, PCR
KD4	7 months	KD	None	Untreated	HTS, PCR
KD5	3.5 weeks	KD	IGIV, ASA, steroid	Treated	HTS, PCR
KD6	4 weeks	KD	IGIV, ASA, steroid, infliximab	Treated	HTS, PCR
KD7	3 weeks	KD	IGIV, ASA	Treated	HTS, PCR
KD8	5 weeks	KD	IGIV, ASA, steroid	Treated	HTS
C1	NA	Enterobacter sepsis, pulmonary hemorrhage, neurologic devastation from herpes simplex virus	NA	Control	HTS
C2	NA	Pneumococcal meningitis, disseminated intravascular coagulation	NA	Control	HTS, PCR
C3	NA	Prematurity, neurologic devastation secondary to Serratia meningitis, chronic lung disease	NA	Control	HTS
C4	NA	Meconium aspiration, pulmonary hemorrhage	NA	Control	HTS
C5	NA	Developmental delay, seizures, fever	NA	Control	HTS
C6	NA	Prematurity, cerebral hemorrhage, bronchopulmonary dysplasia, and pneumonia	NA	Control	HTS, PCR
C7	NA	Cholestasis, renal tubular acidosis, agenesis corpus callosum, dehydration	NA	Control	HTS

Note: HTS, high-throughput RNA sequencing; PCR, real-time reverse transcriptase PCR; IGIV, Immune globulin (intravenous); ASA, Aspirin; KD, Kawasaki disease.
