# Supplementary material for: Upregulated Expression of IL2RB Causes Disorder of Immune Microenvironment in Patients with Kawasaki Disease
Source: Biomed Res Int. 2022 Jul 25;2022:2114699. doi: 10.1155/2022/2114699 (PMC9343205; doi:10.1155/2022/2114699)
Supplement: Supplementary Materials — Supplementary Table 1: clinical data on children whose coronary artery tissues were tested in this study. Supplementary Table 2: the DEGs1 from the comparison between the untreated case group and the control group. Supplementary Table 3: the DEGs2 from the comparison between the treated case group and the control group. Supplementary Table 4: immune cell score matrix estimated by CIBERSORT algorithm. Supplementary Table 5: coexpression analysis of DEGs and immune cell populations. Supplementary Table 6: correlation analysis between the screened 15 core genes and CD4+ memory T cells. [file 2114699.f1.zip › Supplementary Table 2 (1).pdf]

S Table 2. The DEGs1 from the comparison between the untreated case group and the control group.

	logFC	AveExpr	t	P.Value	adj.P.Val	B	Group
IGHG1	6.680993774	6.713647397	7.901406562	1.01E-06	0.007967301	5.626178591	UP
CD74	3.239312272	10.75468793	7.899363968	1.01E-06	0.007967301	5.623484112	UP
PLB1	1.854550062	4.526882061	7.670322706	1.45E-06	0.007967301	5.317245614	UP
APOBEC3G	2.075913328	4.83652205	7.562658381	1.72E-06	0.007967301	5.170459866	UP
EPSTI1	2.796944411	7.037545743	7.500385048	1.90E-06	0.007967301	5.084722882	UP
IGHV3-11	3.218526731	1.707914397	7.497438613	1.90E-06	0.007967301	5.080651036	UP
OAS1	3.17020347	4.873502229	7.422550992	2.15E-06	0.007967301	4.976695805	UP
TRAC	3.263236068	3.892059778	7.358321514	2.38E-06	0.007967301	4.886822455	UP
MX1	3.314091184	6.658977257	7.318393379	2.54E-06	0.007967301	4.830619597	UP
IGHV3-33	2.616849716	1.331021004	7.039778198	4.02E-06	0.009815677	4.431274229	UP
IGKV1-5	4.274181086	2.801960507	6.966593001	4.54E-06	0.009815677	4.324282611	UP
IGLC7	2.709201527	1.08887494	6.933513349	4.80E-06	0.009815677	4.275634894	UP
IL2RB	2.590025931	4.317668456	6.908487595	5.01E-06	0.009815677	4.238712248	UP
LETM1	-1.804322153	7.392566046	-6.897907165	5.10E-06	0.009815677	4.223071125	Down
AOAH	2.620342834	5.970878851	6.883669559	5.22E-06	0.009815677	4.201994571	UP
IFIT2	2.520816975	6.343068402	6.785474061	6.16E-06	0.010658021	4.055723936	UP
IGHV3-49	2.463958175	1.098269731	6.761074055	6.42E-06	0.010658021	4.019131785	UP
IGHGP	4.228866993	1.878667349	6.702800948	7.10E-06	0.011117275	3.931343636	UP
IGKC	3.097141848	1.462225036	6.609449883	8.33E-06	0.012361022	3.789542005	UP
NSFP1	-2.552976434	1.422762739	-6.508394679	9.92E-06	0.013393113	3.634413226	Down
MX2	3.00883559	6.29235996	6.505168141	9.97E-06	0.013393113	3.629432359	UP
SAMD9	2.497790434	8.118879928	6.477552499	1.05E-05	0.013412821	3.586731081	UP
C11orf80	1.928345754	5.661372719	6.403140957	1.19E-05	0.014025417	3.471042229	UP
IGKV3-20	4.709226749	3.146859374	6.402091345	1.19E-05	0.014025417	3.469403825	UP
CCR2	3.283455977	4.779933628	6.270797902	1.50E-05	0.015830414	3.263022325	UP
HMGB1P24	-2.396020116	2.160071783	-6.237059145	1.60E-05	0.015830414	3.209527796	Down
HLA-DOA	3.487425226	5.247999685	6.234734232	1.60E-05	0.015830414	3.205834604	UP
IGLV3-1	3.357747543	2.037859657	6.234552339	1.60E-05	0.015830414	3.205545624	UP
IGLV3-21	2.700380716	1.573380735	6.224099278	1.63E-05	0.015830414	3.188929295	UP
IGKV3-15	3.864837078	1.797074093	6.206912192	1.68E-05	0.015830414	3.16156927	UP

IGHG3	5.503248585	4.43055165	6.021046034	2.35E-05	0.020483347	2.862583267	UP
IGHV3-15	3.620431994	2.291569159	6.01532001	2.37E-05	0.020483347	2.853282283	UP
TAGAP	2.527660738	5.21738863	6.009774062	2.40E-05	0.020483347	2.844268687	UP
MS4A4E	2.960693997	4.743356577	5.976867385	2.54E-05	0.021098493	2.790683403	UP
SP110	1.71013722	7.322607653	5.94682204	2.69E-05	0.021297075	2.741603101	UP
ANKRD36BP2	3.042931307	5.974493443	5.878891071	3.04E-05	0.021297075	2.630093245	UP
ARHGEF6	1.394725935	9.029766948	5.855552254	3.17E-05	0.021297075	2.591609262	UP
TLR7	4.241626501	4.340572964	5.843341851	3.24E-05	0.021297075	2.571440071	UP
MIR1276	-1.770133595	0.90292575	-5.832166394	3.31E-05	0.021297075	2.552959276	Down
HOOK2	-2.058762315	4.972936539	-5.824959028	3.35E-05	0.021297075	2.541029788	Down
SULF1	2.953794888	9.861383695	5.818511648	3.39E-05	0.021297075	2.530351107	UP
SLC27A5	-2.602129436	2.313255756	-5.804466447	3.48E-05	0.021297075	2.507065075	Down
CD8B	3.322114275	2.215395653	5.80137922	3.50E-05	0.021297075	2.501942388	UP
ITGA4	3.382732279	7.785374886	5.789623194	3.58E-05	0.021297075	2.482421372	UP
OAS2	3.180674474	6.834328911	5.78897409	3.58E-05	0.021297075	2.48134288	UP
IGHV5-51	2.985986238	1.675724212	5.785641862	3.60E-05	0.021297075	2.475805282	UP
TOMM40	-1.969374061	5.472028837	-5.781185028	3.63E-05	0.021297075	2.468395988	Down
HLA-DMB	3.398558005	6.446501636	5.772629809	3.69E-05	0.021297075	2.454164355	UP
IKZF3	3.709655924	6.757793871	5.756738663	3.80E-05	0.021297075	2.427698185	UP
ZDHHC4P1	2.957144314	3.740340815	5.755013321	3.81E-05	0.021297075	2.424822246	UP
IGLC3	4.63617265	3.673178054	5.746793982	3.87E-05	0.021297075	2.411115039	UP
CIITA	2.683189439	8.247941631	5.738911757	3.93E-05	0.021297075	2.397959858	UP
HLA-DPA1	4.656266065	7.904753382	5.715053898	4.10E-05	0.021368642	2.358081264	UP
DCDC2C	2.166044052	0.906996041	5.697973204	4.23E-05	0.021368642	2.329474893	UP
ZBP1	2.954444166	2.326871118	5.691819012	4.28E-05	0.021368642	2.319156586	UP
LPAR5	1.844247416	3.900207907	5.691563673	4.29E-05	0.021368642	2.318728346	UP
ICOS	3.048506152	3.5006888	5.670697328	4.45E-05	0.021368642	2.283697452	UP
SLAMF7	4.251913025	4.489433975	5.664989147	4.50E-05	0.021368642	2.274102367	UP
IGKV3-11	3.642166258	2.440560802	5.663571195	4.51E-05	0.021368642	2.271718079	UP
ARHGAP15	2.39345551	7.089020139	5.651712731	4.61E-05	0.021368642	2.251765557	UP
TLR5	2.221609404	4.855221949	5.645778998	4.66E-05	0.021368642	2.241773351	UP
TAPSAR1	1.620337497	4.580320163	5.641901381	4.70E-05	0.021368642	2.23524056	UP
MNDA	2.448705383	6.060912464	5.569254585	5.38E-05	0.024070396	2.112410395	UP

CD2	3.378607837	4.194988308	5.549861805	5.58E-05	0.02419708	2.079481029	UP
MLX	-1.243576153	5.830170204	-5.546788259	5.61E-05	0.02419708	2.074256676	Down
SAMD3	2.661790651	4.5329197	5.524816064	5.84E-05	0.02419708	2.036865787	UP
STARD5	1.748368341	4.784987352	5.508036489	6.03E-05	0.02419708	2.008260688	UP
ANKRD44-IT1	2.118643668	5.828043717	5.500688152	6.11E-05	0.02419708	1.995719774	UP
GGN	-2.567047602	2.052547964	-5.498727407	6.13E-05	0.02419708	1.992372082	Down
MS4A6A	3.57768421	8.216329506	5.490312447	6.23E-05	0.02419708	1.977997977	UP
KCNG2	-2.016044726	1.330090531	-5.480572355	6.35E-05	0.02419708	1.961346626	Down
IGLV6-57	2.620840494	1.082304603	5.477516255	6.38E-05	0.02419708	1.956118989	UP
MCAT	-1.416990072	3.589838443	-5.476906196	6.39E-05	0.02419708	1.955075274	Down
GPR174	2.644234486	3.184431251	5.472215487	6.45E-05	0.02419708	1.94704829	UP
TRMT61A	-1.808861718	4.37098033	-5.464803359	6.54E-05	0.02419708	1.934357341	Down
C3orf27	-2.61865026	1.898437275	-5.462296312	6.57E-05	0.02419708	1.930062887	Down
RPS26P56	-1.773066806	1.375067983	-5.459213331	6.61E-05	0.02419708	1.924780556	Down
CERKL	3.026077448	5.730778479	5.44233615	6.82E-05	0.024656797	1.895837509	UP
IL20RB	-2.213778105	2.282978959	-5.408299885	7.27E-05	0.025096416	1.837334874	Down
GBP5	3.651749325	6.587994419	5.40199757	7.36E-05	0.025096416	1.826482792	UP
CLEC10A	2.565393062	4.070043381	5.400289645	7.38E-05	0.025096416	1.823540836	UP
SORL1	2.54048927	8.805500966	5.400175747	7.38E-05	0.025096416	1.823344628	UP
BTN2A2	2.293178452	5.746133518	5.399968116	7.39E-05	0.025096416	1.822986944	UP
SNORD121A	-2.062643016	1.861188946	-5.366338079	7.87E-05	0.025956262	1.76496599	Down
GAPT	3.113742467	3.580181122	5.365286233	7.89E-05	0.025956262	1.763148499	UP
SNORD96B	-2.324110566	2.075667879	-5.363333341	7.92E-05	0.025956262	1.759773638	Down
DMP1	2.383622479	2.144153631	5.345788594	8.18E-05	0.026243292	1.729428066	UP
CD8A	2.460322536	4.217867318	5.338413703	8.30E-05	0.026243292	1.716658495	UP
LOC10013261	2.626255362	1.400837796	5.337761205	8.31E-05	0.026243292	1.715528305	UP
LCP2	2.867246987	7.564711471	5.333509182	8.37E-05	0.026243292	1.70816181	UP
RNU1-19P	-1.855088639	1.256801369	-5.309043759	8.77E-05	0.026997623	1.665723406	Down
RPL23AP7	1.450317594	4.356036024	5.303860882	8.86E-05	0.026997623	1.656721514	UP
SLC15A3	1.566293951	5.85339459	5.30128746	8.90E-05	0.026997623	1.652250365	UP
TRBC2	2.961951636	3.910814377	5.271557216	9.42E-05	0.028263574	1.600524359	UP
IGHM	5.325358013	6.208933059	5.250634692	9.80E-05	0.029102837	1.564043609	UP
AMICA1	3.407010026	6.094638158	5.244119809	9.93E-05	0.029159548	1.552670953	UP

IGLV1-44	3.281163738	1.563703964	5.210491372	0.000105839	0.029558449	1.493868246	UP
IGHV1-2	4.457200482	2.53961816	5.201377137	0.000107699	0.029558449	1.477902498	UP
XAF1	2.492447849	8.910175029	5.201032565	0.00010777	0.029558449	1.477298661	UP
RPL15P18	-1.667319906	1.55435299	-5.200316294	0.000107918	0.029558449	1.47604339	Down
BLNK	2.878765816	4.097887092	5.19995729	0.000107992	0.029558449	1.475414206	UP
P2RX7	1.807280405	4.604906247	5.197741896	0.000108451	0.029558449	1.471531123	UP
COMTD1	-2.435887525	2.877908323	-5.195517675	0.000108913	0.029558449	1.467631845	Down
STAT1	2.22754756	10.33917852	5.19442803	0.00010914	0.029558449	1.465721328	UP
MIR635	-1.862056461	2.470778338	-5.190087602	0.00011005	0.029558449	1.458109365	Down
CD3E	3.84975333	3.945806434	5.1602381	0.000116525	0.031002276	1.405687035	UP
TLR6	2.295649836	5.833938028	5.144480886	0.000120101	0.031304399	1.377961857	UP
EIF4A1P10	-1.555441184	0.954648538	-5.135323368	0.000122231	0.031304399	1.36183256	Down
RPS27P15	-1.685276917	1.668035864	-5.134769293	0.000122361	0.031304399	1.36085627	Down
ANXA2R	1.494955099	3.60361544	5.133893405	0.000122567	0.031304399	1.359312852	UP
FCRL5	4.642822863	4.810302935	5.13116556	0.000123211	0.031304399	1.354505361	UP
IFNG-AS1	3.274441193	3.104556805	5.110467111	0.000128211	0.03137712	1.317992171	UP
TAP2	1.679198238	7.231763472	5.107563086	0.00012893	0.03137712	1.312864418	UP
DOCK10	2.574938708	8.746338304	5.106074651	0.000129299	0.03137712	1.310235762	UP
BTBD19	1.930602434	5.974828109	5.100844182	0.000130607	0.03137712	1.300995972	UP
C5orf58	2.084263856	4.037856731	5.100342918	0.000130733	0.03137712	1.300110268	UP
TRMT1	-1.301092882	4.970340907	-5.093625635	0.000132435	0.03137712	1.288237791	Down
SFRP4	4.714504282	6.397291165	5.090751979	0.00013317	0.03137712	1.283156785	UP
PARP9	1.879061204	8.328905887	5.08845983	0.000133759	0.03137712	1.279103121	UP
IFIH1	1.964183965	6.572902434	5.087308397	0.000134056	0.03137712	1.27706653	UP
FCRL3	3.502713009	3.043326948	5.085117141	0.000134623	0.03137712	1.273190243	UP
TELO2	-1.610535996	5.270563652	-5.076788023	0.000136801	0.031623444	1.258449999	Down
CARD16	1.413037306	4.273113553	5.070723252	0.00013841	0.031735237	1.24771086	UP
POR	-1.766072736	6.639353649	-5.065766325	0.000139739	0.031781687	1.238929576	Down
PFN1P3	-1.693484917	1.770479281	-5.05591485	0.000142421	0.032075852	1.221467253	Down
IGHA2	2.576500576	2.369193538	5.050646963	0.000143877	0.032075852	1.212124021	UP
CD69	3.042225825	5.828155308	5.043939022	0.000145753	0.032075852	1.200221054	UP
GGTA1P	2.717935324	5.988718218	5.041345813	0.000146484	0.032075852	1.195617826	UP
CLUH	-2.171471409	7.16002871	-5.040515872	0.00014672	0.032075852	1.194144391	Down

RPS2P7	-1.5618099	0.884677401	-5.033240436	0.000148797	0.032160309	1.181223842	Down
TMED1	-1.320655703	4.532957661	-5.029988792	0.000149735	0.032160309	1.175446818	Down
CCR5	3.012499542	4.445125739	5.027260805	0.000150527	0.032160309	1.170599011	UP
LONP1	-1.789520225	6.869501657	-5.013084822	0.000154712	0.032805905	1.145390753	Down
ANKRD22	3.382942568	2.75419333	5.00779696	0.000156304	0.032896069	1.135980552	UP
ABCB6	-2.214975057	3.334980418	-4.996719203	0.000159693	0.033360467	1.116254228	Down
BTN3A1	2.180380708	7.038170478	4.99110741	0.000161439	0.033477213	1.106254776	UP
CD3G	3.300026884	4.10956368	4.979029768	0.000165264	0.034017175	1.084719399	UP
RNY4P6	-2.011986125	2.504057314	-4.973097925	0.000167176	0.034017175	1.074135149	Down
IGHV1-18	3.506861095	2.248665232	4.971604079	0.000167661	0.034017175	1.071468907	UP
IL1RL1	-3.885336016	8.419930965	-4.962933937	0.000170507	0.034107644	1.05598828	Down
IGLV3-25	2.769523872	1.654757216	4.962831359	0.000170541	0.034107644	1.055805064	UP
MIR1913	-1.718622061	1.020763659	-4.959234931	0.000171736	0.034107644	1.049380553	Down
FCGR2C	3.501226948	6.459861428	4.954527325	0.000173313	0.034180197	1.040968425	UP
NECAB3	-1.624124113	5.644570036	-4.93889581	0.000178658	0.034851813	1.013014586	Down
EEF1B2P1	2.109722695	2.626642349	4.937367512	0.00017919	0.034851813	1.01027976	UP
IGHV1-69	3.839029348	2.256241528	4.926693961	0.000182949	0.035184841	0.991171117	UP
BTN3A3	1.755375038	6.548041524	4.92543546	0.000183397	0.035184841	0.988917038	UP
IGKV4-1	3.06787032	2.450273025	4.908354861	0.000189598	0.035718397	0.958303325	UP
TBRG4	-1.342506189	6.403712367	-4.904908712	0.000190875	0.035718397	0.952122039	Down
IGKJ3	2.236536953	1.022132504	4.901352819	0.000192202	0.035718397	0.94574225	UP
HLA-DMA	2.207642954	5.955856802	4.899738006	0.000192808	0.035718397	0.942844486	UP
TPP1	1.69155052	8.823316905	4.89853703	0.00019326	0.035718397	0.940689123	UP
ZNF683	2.31635463	1.866692362	4.89716499	0.000193778	0.035718397	0.938226521	UP
CDC42EP4	-1.773166049	7.752672156	-4.886215422	0.000197959	0.03625221	0.918564785	Down
FYB	3.824024461	8.795706434	4.874175157	0.000202665	0.036874483	0.896926242	UP
POU2AF1	3.144056225	3.946497714	4.86524307	0.00020623	0.037282622	0.880861341	UP
TRIM22	1.707063389	9.401787032	4.854042519	0.000210792	0.037864646	0.860701704	UP
BCL11B	3.576479872	5.177978835	4.846202874	0.000214047	0.037978236	0.846581557	UP
LOC10192937:	-2.168904557	2.187682579	-4.846033872	0.000214117	0.037978236	0.846277075	Down
KIAA1211	2.269483099	3.720327165	4.827129562	0.000222184	0.03888827	0.812194918	UP
IGHG2	4.664639839	3.484390631	4.824659025	0.000223261	0.03888827	0.807737432	UP
APOBEC3F	1.656941809	3.523436113	4.823297361	0.000223857	0.03888827	0.805280302	UP

IGHV3-30	2.852230292	1.622831638	4.821231745	0.000224764	0.03888827	0.801552433	UP
S1PR2	-1.363747753	6.291381064	-4.815142069	0.00022746	0.039114829	0.790559049	Down
PRSS45	-3.553996432	2.395173787	-4.810451074	0.00022956	0.039236669	0.782087391	Down
OLFML2B	2.386727577	6.388397717	4.803491028	0.000232712	0.039257485	0.769512775	UP
MAP4K1	2.231622174	3.433428902	4.797807882	0.000235319	0.039257485	0.759240535	UP
TTLL12	-1.737292507	5.009689683	-4.797692264	0.000235373	0.039257485	0.759031513	Down
SETP6	-2.973064042	2.878775622	-4.796473884	0.000235936	0.039257485	0.756828748	Down
HMGB1P19	-1.304776786	1.017572411	-4.79494963	0.000236642	0.039257485	0.754072714	Down
ST6GALNAC4	-2.035712479	1.538478964	-4.789264755	0.000239295	0.03927129	0.743791175	Down
SNX20	2.07605763	4.461935565	4.788806833	0.00023951	0.03927129	0.742962809	UP
MCF2L-AS1	-1.706166005	1.119791095	-4.778277136	0.00024451	0.039859389	0.723907615	Down
MTCP1	-1.649035522	3.323845034	-4.771955844	0.000247563	0.040125174	0.712461494	Down
PLXNC1	1.778705591	7.994054724	4.765449432	0.000250747	0.040238618	0.700674926	UP
LINC01094	2.995155788	4.298065967	4.764698921	0.000251117	0.040238618	0.69931501	UP
CXCL14	3.772380648	4.619037935	4.757043474	0.000254922	0.04049029	0.685439421	UP
SH2D1A	2.828994623	4.020618412	4.754867513	0.000256015	0.04049029	0.68149413	UP
MIAT	2.469804018	6.396589053	4.75292431	0.000256995	0.04049029	0.67797036	UP
FILIP1L	1.436083218	10.14789217	4.748542035	0.000259218	0.04061373	0.670021894	UP
GPR56	-1.42901806	6.67818849	-4.74572218	0.000260659	0.04061391	0.664906045	Down
IGHV3-23	3.874287302	2.53913061	4.737885893	0.000264708	0.04101812	0.65068409	UP
SLAMF6	2.752511798	4.467694008	4.732964027	0.000267284	0.041190951	0.641747589	UP
ASB6	-1.237303432	4.649919657	-4.726709527	0.000270595	0.041424681	0.630387152	Down
RALYL	1.990830865	0.890394977	4.718194551	0.00027517	0.041424681	0.614913145	UP
TRAF5	1.959418084	8.472213118	4.716974994	0.000275831	0.041424681	0.612696154	UP
JMJD4	-1.520183411	3.845732251	-4.716782525	0.000275936	0.041424681	0.612346253	Down
COASY	-1.438291817	5.182988607	-4.716398248	0.000276145	0.041424681	0.611647642	Down
CLDN1	2.706160313	4.346591999	4.712348742	0.000278357	0.041535518	0.604284594	UP
DSPP	2.027884114	2.270864329	4.704552978	0.000282666	0.041956517	0.590104251	UP
IGLV3-19	3.061561884	1.953393012	4.70157814	0.000284328	0.041982305	0.58469113	UP
TRHR	-2.009876032	2.338012576	-4.689856938	0.000290976	0.042740191	0.563352381	Down
FAM46C	2.988777217	6.917639074	4.684161855	0.000294264	0.042805299	0.552978351	UP
C4orf50	1.583150558	0.60533876	4.681790436	0.000295644	0.042805299	0.548657481	UP
FAM26F	2.024901109	3.349755167	4.68082679	0.000296207	0.042805299	0.546901465	UP

KCNA3	2.395047416	4.633516257	4.678635725	0.000297491	0.042805299	0.542908356	UP
CXorf21	2.543529816	3.851779237	4.670767965	0.000302148	0.042986822	0.528565013	UP
THOC6	-2.813838062	3.429780641	-4.668842774	0.000303299	0.042986822	0.52505416	Down
CD1C	2.804062225	1.752398979	4.665122712	0.000305535	0.042986822	0.518268865	UP
EPS8L3	2.496578755	1.862603052	4.664225718	0.000306077	0.042986822	0.516632523	UP
ITGAL	2.396219612	5.536282426	4.660332654	0.00030844	0.042986822	0.509529498	UP
TOR4A	-2.036588519	4.562128078	-4.659613914	0.000308879	0.042986822	0.508217937	Down
NAGK	1.536559796	7.845702824	4.658723937	0.000309422	0.042986822	0.506593815	UP
RNA5SP321	1.505742729	1.96078512	4.653668243	0.000312529	0.043205623	0.497365886	UP
ESRRA	-1.866397723	6.075511723	-4.651157953	0.000314084	0.043208728	0.492782845	Down
LINC00426	2.375978323	3.263964592	4.640978144	0.00032047	0.043873244	0.47418994	UP
AURKAIP1	-1.941219086	4.554747742	-4.637617874	0.000322607	0.043934495	0.468049904	Down
HLA-DPB1	3.233691405	6.46754536	4.635388481	0.000324033	0.043934495	0.463975528	UP
SLFN12L	2.003811181	4.552427044	4.625798933	0.000330241	0.044476945	0.446443323	UP
AURKC	-2.101383227	1.98543286	-4.62292273	0.000332126	0.044476945	0.441182785	Down
IGHV4-39	2.703757021	2.001288505	4.6203307	0.000333835	0.044476945	0.436441171	UP
RSAD2	3.020239888	5.441095541	4.618568818	0.000335002	0.044476945	0.433217708	UP
P2RY13	2.580139972	4.338910337	4.617188174	0.000335919	0.044476945	0.430691489	UP
MIR601	-1.701996043	1.198465378	-4.612067083	0.000339344	0.044720409	0.421319305	Down
MAPK12	-1.254325741	5.256709302	-4.609210195	0.00034127	0.044765042	0.416089558	Down
SUSD3	2.555318613	2.506782081	4.604392094	0.000344543	0.044945576	0.407267536	UP
MTA1	-1.410119881	6.433343308	-4.602506195	0.000345833	0.044945576	0.403813698	Down
STAMBPL1	1.689179111	6.378759038	4.59360471	0.00035199	0.045440093	0.38750601	UP
TRGV10	1.648004032	1.458734967	4.592358473	0.000352861	0.045440093	0.385222159	UP
SIAH2-AS1	-1.528240586	1.743083293	-4.589313106	0.000354998	0.045507549	0.379640482	Down
RPL7P18	-2.023576072	1.953211871	-4.578676032	0.000362569	0.046267735	0.360136141	Down
IL2RG	2.525314788	4.773962832	4.575368683	0.000364956	0.046291986	0.354069114	UP
RAB30	1.586037653	8.148352665	4.573872477	0.000366042	0.046291986	0.351324058	UP
STX7	1.249957177	9.464391356	4.567085551	0.000371007	0.04666125	0.338869073	UP
ADAMDEC1	3.235912212	3.856361344	4.565372994	0.000372271	0.04666125	0.335725468	UP
CCL5	2.6834974	4.486802784	4.556873171	0.000378609	0.047245685	0.320118164	UP
TP53INP1	1.68699674	9.143376624	4.541468981	0.000390379	0.048128061	0.291812657	UP
LOC10028947	-1.487191489	1.145046458	-4.540219848	0.000391349	0.048128061	0.2895162	Down

FAP	3.55786712	6.329676488	4.538735622	0.000392506	0.048128061	0.286787317	UP
PARP15	2.013560363	6.892434943	4.536334564	0.000394385	0.048149059	0.282372239	UP
UBE2L6	1.417482407	6.31811081	4.531223677	0.000398414	0.048431337	0.272972211	UP
TPGS1	-2.189042212	2.338529484	-4.527585628	0.000401308	0.048573755	0.26627931	Down
MID1IP1-AS1	-1.888430695	1.468633674	-4.521617713	0.000406102	0.048943957	0.255297041	Down
CDS1	-3.024779313	3.925864545	-4.518797301	0.000408388	0.048986031	0.250105516	Down
HERPUD1	1.404669329	9.310562144	4.516910073	0.000409925	0.048986031	0.24663122	UP
PARP14	1.722158639	10.77146308	4.509281916	0.000416198	0.049252218	0.232584222	UP
APOBEC3D	1.480147842	3.435077366	4.509013921	0.00041642	0.049252218	0.232090604	UP
MTHFR	-1.501364109	7.992729238	-4.505819056	0.000419078	0.049252218	0.22620541	Down
CD96	2.503908928	5.491327029	4.505747574	0.000419138	0.049252218	0.226073722	UP
IGJ	5.181241331	6.659998363	4.498257633	0.000425439	0.049697379	0.212272305	UP
HLA-DRA	3.376733157	9.006381226	4.49547534	0.000427804	0.049697379	0.207143956	UP
TPM3P7	-1.914299999	1.586137971	-4.493167975	0.000429776	0.049697379	0.202890372	Down
GPR114	2.059647383	2.857656256	4.492935024	0.000429975	0.049697379	0.2024609	UP
PRND	4.197781704	2.387620718	4.490352818	0.000432194	0.049749919	0.197699915	UP
CCDC11P1	1.49549491	0.354553109	4.48683317	0.000435237	0.049769921	0.191209367	UP
TWIST2	-1.493694884	3.14368808	-4.486072752	0.000435897	0.049769921	0.189806915	Down

Note: DEGs, differentially expressed genes.
