# Supplementary material for: Upregulated Expression of IL2RB Causes Disorder of Immune Microenvironment in Patients with Kawasaki Disease
Source: Biomed Res Int. 2022 Jul 25;2022:2114699. doi: 10.1155/2022/2114699 (PMC9343205; doi:10.1155/2022/2114699)
Supplement: Supplementary Materials — Supplementary Table 1: clinical data on children whose coronary artery tissues were tested in this study. Supplementary Table 2: the DEGs1 from the comparison between the untreated case group and the control group. Supplementary Table 3: the DEGs2 from the comparison between the treated case group and the control group. Supplementary Table 4: immune cell score matrix estimated by CIBERSORT algorithm. Supplementary Table 5: coexpression analysis of DEGs and immune cell populations. Supplementary Table 6: correlation analysis between the screened 15 core genes and CD4+ memory T cells. [file 2114699.f1.zip › Supplementary Table 3 (1).pdf]

S Table 3. The DEGs2 from the comparison between the treated case group and the control group.

	logFC	AveExpr	t	P.Value	adj.P.Val	B	Group
TRAC	3.738242931	3.892059778	8.429421842	4.52E-07	0.010117016	6.250630325	UP
ADAMDEC1	5.586644189	3.856361344	7.881893214	1.04E-06	0.010117016	5.55788337	UP
PLB1	1.821382237	4.526882061	7.533142307	1.80E-06	0.010117016	5.09258025	UP
CD8A	3.408259268	4.217867318	7.395249084	2.24E-06	0.010117016	4.903288186	UP
IL2RB	2.754151331	4.317668456	7.346266335	2.43E-06	0.010117016	4.835312834	UP
SORL1	3.455346437	8.805500966	7.344836384	2.44E-06	0.010117016	4.833322623	UP
MNDA	3.221136846	6.060912464	7.326047173	2.51E-06	0.010117016	4.807141069	UP
TRBC2	3.964825793	3.910814377	7.056430552	3.91E-06	0.013785844	4.425134488	UP
CD3G	4.571002713	4.10956368	6.896658536	5.11E-06	0.014392648	4.193145781	UP
AOAH	2.586648801	5.970878851	6.795154963	6.06E-06	0.014392648	4.043574117	UP
CD74	2.779261917	10.75468793	6.77748843	6.25E-06	0.014392648	4.017367062	UP
SAMD3	3.207655789	4.5329197	6.657814441	7.66E-06	0.014392648	3.838474793	UP
CD8B	3.774820655	2.215395653	6.591936428	8.58E-06	0.014392648	3.738982023	UP
LOC101929241	2.626440673	0.993308234	6.546203145	9.29E-06	0.014392648	3.669487975	UP
TRAV12-1	1.856121826	0.590522488	6.531583061	9.53E-06	0.014392648	3.647198504	UP
TRGV10	2.32860993	1.458734967	6.488947436	1.03E-05	0.014392648	3.581993693	UP
EVI2B	2.717870984	7.698287619	6.472789276	1.06E-05	0.014392648	3.55720302	UP
LOC101060038	3.524763957	2.974845699	6.4495444	1.10E-05	0.014392648	3.521463195	UP
CARD16	1.797179179	4.273113553	6.449226933	1.10E-05	0.014392648	3.520974454	UP
SELL	4.712114234	5.649524499	6.431908462	1.13E-05	0.014392648	3.494287209	UP
IL2RG	3.528119758	4.773962832	6.392252056	1.21E-05	0.014392648	3.432989526	UP
CCR2	3.345822106	4.779933628	6.389905756	1.22E-05	0.014392648	3.429354588	UP
CD2	3.889591452	4.194988308	6.389227776	1.22E-05	0.014392648	3.428304076	UP
PTPN22	3.286279928	5.710589566	6.387331292	1.22E-05	0.014392648	3.425365118	UP
TRAV21	1.610623489	0.526122167	6.282996843	1.47E-05	0.014842443	3.262755775	UP
TAGAP	2.637602264	5.21738863	6.271171379	1.50E-05	0.014842443	3.244210917	UP
SH2D1A	3.725181692	4.020618412	6.261144954	1.53E-05	0.014842443	3.2284691	UP

THEMIS	4.723043725	4.047777411	6.242775287	1.58E-05	0.014842443	3.199584715	UP
TRAV29DV5	1.595890153	0.515565795	6.240070993	1.59E-05	0.014842443	3.195327754	UP
IGLV3-1	3.352289281	2.037859657	6.224417622	1.63E-05	0.014842443	3.170663116	UP
IKZF2	2.494521414	8.056315081	6.208200425	1.68E-05	0.014842443	3.145067097	UP
BCL11B	4.58063832	5.177978835	6.206857968	1.68E-05	0.014842443	3.142946303	UP
FCGR3B	3.933040378	4.341478957	6.162276251	1.82E-05	0.015349722	3.072346669	UP
ITGA4	3.5955925	7.785374886	6.153938301	1.85E-05	0.015349722	3.059106048	UP
GBP5	4.073329046	6.587994419	6.025636386	2.33E-05	0.018768338	2.853910765	UP
CD69	3.622494424	5.828155308	6.006010741	2.41E-05	0.018904337	2.822283155	UP
CD96	3.315844173	5.491327029	5.966813199	2.59E-05	0.019566018	2.758924528	UP
IGHV3-33	2.214384936	1.331021004	5.957078353	2.64E-05	0.019566018	2.743149985	UP
GPR174	2.871064791	3.184431251	5.941638421	2.71E-05	0.019605351	2.718098853	UP
RHOH	3.420505185	4.892460479	5.924052943	2.80E-05	0.019620112	2.6895189	UP
CAMK4	3.669989484	6.786256209	5.902180818	2.91E-05	0.019620112	2.653901488	UP
LINC00426	3.020758924	3.263964592	5.900422578	2.92E-05	0.019620112	2.651034897	UP
CD3E	4.388506865	3.945806434	5.88238736	3.02E-05	0.019803432	2.621601501	UP
IGHV3-11	2.492958767	1.707914397	5.807254961	3.46E-05	0.022200436	2.498414053	UP
MIR1276	-1.752243274	0.90292575	-5.773222072	3.69E-05	0.022545786	2.442311177	Down
PLXNC1	2.150566149	7.994054724	5.761725991	3.77E-05	0.022545786	2.423317552	UP
SLAMF6	3.334870491	4.467694008	5.73433403	3.96E-05	0.022545786	2.377974834	UP
IKZF3	3.69504532	6.757793871	5.734065554	3.96E-05	0.022545786	2.377529819	UP
TRBV29-1	1.552343172	0.534391281	5.725779799	4.02E-05	0.022545786	2.363789922	UP
LCP2	3.076145231	7.564711471	5.722091229	4.05E-05	0.022545786	2.357669767	UP
ICOS	3.074218535	3.5006888	5.718526375	4.08E-05	0.022545786	2.351752802	UP
CERKL	3.170043198	5.730778479	5.7012555	4.21E-05	0.022810862	2.323057502	UP
CD48	3.033905504	4.743007889	5.689380714	4.30E-05	0.022810862	2.303299871	UP
SIRPG	3.468133303	1.891575685	5.677838638	4.40E-05	0.022810862	2.284074093	UP
C5orf58	2.317594192	4.037856731	5.671318961	4.45E-05	0.022810862	2.273204735	UP
LEF1	4.411784901	5.860973589	5.657307732	4.57E-05	0.022992226	2.249822685	UP
APOBEC3G	1.546744524	4.83652205	5.634869376	4.76E-05	0.023548213	2.212311916	UP

IGLV3-21	2.421873079	1.573380735	5.582167874	5.25E-05	0.024056324	2.123893683	UP
PTPRC	3.656825974	10.43320235	5.581998094	5.25E-05	0.024056324	2.123608126	UP
AQP9	3.543951079	5.893168421	5.581408839	5.26E-05	0.024056324	2.122617012	UP
ANKRD44-IT1	2.146969731	5.828043717	5.574231827	5.33E-05	0.024056324	2.110540997	UP
FYB	4.370393475	8.795706434	5.570587614	5.36E-05	0.024056324	2.104406133	UP
TLR6	2.482463966	5.833938028	5.563125624	5.44E-05	0.024056324	2.091837656	UP
LPXN	1.954126043	6.076365586	5.561121897	5.46E-05	0.024056324	2.088461212	UP
TRAJ21	3.097228198	1.920930214	5.55101374	5.56E-05	0.024137168	2.07141846	UP
CD226	2.776882325	5.401568761	5.522840187	5.86E-05	0.02505621	2.023831638	UP
GIMAP2	1.883546892	5.792569558	5.503163548	6.08E-05	0.025608083	1.990522533	UP
LY75	2.455541553	5.296755573	5.478779861	6.37E-05	0.026145439	1.949160997	UP
ARHGAP15	2.319209461	7.089020139	5.476394102	6.40E-05	0.026145439	1.94510909	UP
S1PR2	-1.544683065	6.291381064	-5.453991321	6.67E-05	0.026339365	1.907017506	Down
CD3D	3.423745482	2.778902979	5.446589174	6.77E-05	0.026339365	1.894414413	UP
IGKC	2.55212279	1.462225036	5.446352962	6.77E-05	0.026339365	1.89401209	UP
BTN3A3	1.939638161	6.548041524	5.442462362	6.82E-05	0.026339365	1.887384294	UP
GPR141	2.665790659	3.50963007	5.433456736	6.93E-05	0.026427418	1.872033825	UP
IGHV5-51	2.798462187	1.675724212	5.42229558	7.08E-05	0.026517171	1.852991714	UP
WIPF1	1.837327168	9.861533121	5.412623154	7.21E-05	0.026517171	1.83647394	UP
C16orf54	2.428000882	3.612399253	5.408491529	7.27E-05	0.026517171	1.829413884	UP
LCK	3.106451894	3.813525083	5.403713543	7.33E-05	0.026517171	1.82124605	UP
TRAJ11	2.926923462	1.226854027	5.393589027	7.48E-05	0.026686393	1.803926839	UP
RAB39B	2.516767549	3.215765302	5.354650819	8.05E-05	0.028365069	1.737171514	UP
GPR171	2.537444522	4.229819933	5.294957741	9.01E-05	0.031372229	1.634384948	UP
HLA-DOA	2.952924085	5.247999685	5.279166057	9.29E-05	0.031887158	1.607102707	UP
LOC101927156	3.008336462	2.43399827	5.273567149	9.38E-05	0.031887158	1.597420829	UP
CXCR4	4.51068989	7.58879706	5.266370649	9.51E-05	0.031942061	1.584969408	UP
DOCK10	2.650139126	8.746338304	5.25519624	9.72E-05	0.032245216	1.565619991	UP
TMEM154	2.067472419	5.905308172	5.224666751	0.000103012	0.033780887	1.512660515	UP
NELL2	3.646555556	3.125901898	5.211875684	0.00010556	0.03421838	1.490430635	UP

LOC101927095	1.810639866	0.667949845	5.198867663	0.000108217	0.034681223	1.467798849	UP
IKZF1	3.796968213	7.82178843	5.191115321	0.000109834	0.034803824	1.454299177	UP
ITK	3.963244869	6.173552733	5.152437024	0.000118281	0.037064129	1.386814081	UP
TRAJ9	2.930976532	1.605048178	5.13542929	0.000122206	0.037490577	1.357070103	UP
SAMD9	1.977889644	8.118879928	5.129287003	0.000123656	0.037490577	1.346317826	UP
LY9	3.292496454	3.95026509	5.120412152	0.000125783	0.037490577	1.330772444	UP
TMEM156	3.27779848	3.287396726	5.119537701	0.000125995	0.037490577	1.32924012	UP
PKDCC	-1.452214475	6.934595053	-5.11832317	0.000126289	0.037490577	1.327111679	Down
TRAF3IP3	3.359356832	6.429786111	5.112181973	0.000127789	0.037540726	1.3163461	UP
ADAM8	2.803618432	5.110476612	5.090172133	0.000133318	0.038564336	1.277717985	UP
RSPO4	-2.758692592	1.998969592	-5.087491636	0.000134008	0.038564336	1.273008856	Down
EPSTI1	1.891449533	7.037545743	5.07217796	0.000138022	0.039037508	1.24608588	UP
ITGAL	2.604136346	5.536282426	5.064703414	0.000140026	0.039037508	1.232932721	UP
UBASH3A	2.849004075	2.657495827	5.062669589	0.000140577	0.039037508	1.229352368	UP
CDC42EP4	-1.836381569	7.752672156	-5.060414926	0.000141189	0.039037508	1.225382563	Down
IGHV3-15	3.032803879	2.291569159	5.038980401	0.000147155	0.040292024	1.187606582	UP
COMP	4.416251136	4.447067834	5.008244463	0.000156168	0.041968085	1.133324778	UP
LINC00861	3.608118989	5.463429359	5.007963896	0.000156253	0.041968085	1.132828667	UP
FLT3	2.923634111	3.811341182	5.000348253	0.000158574	0.041970728	1.11935814	UP
IGHV3-49	1.820474071	1.098269731	4.99536077	0.000160114	0.041970728	1.110531912	UP
IGKV3-11	3.208269003	2.440560802	4.988860646	0.000162143	0.041970728	1.099023602	UP
CXCR2	2.533741833	2.929059112	4.988630556	0.000162216	0.041970728	1.098616125	UP
CYTIP	4.033915837	7.530886004	4.98255345	0.000164138	0.042082045	1.087851254	UP
LETM1	-1.29637548	7.392566046	-4.956031658	0.000172807	0.042549379	1.040811256	Down
XCL1	2.590489963	1.672897505	4.954634299	0.000173277	0.042549379	1.038330163	UP
AMICA1	3.218561555	6.094638158	4.954057159	0.000173471	0.042549379	1.03730534	UP
OSGIN1	-1.617292155	3.228391028	-4.953358573	0.000173707	0.042549379	1.036064803	Down
LOC100996286	2.996893043	1.365004539	4.95215078	0.000174115	0.042549379	1.033919868	UP
IGHV4OR15-8	1.712197542	0.504981373	4.946619193	0.000175996	0.042549379	1.024093696	UP
TAPSAR1	1.420213505	4.580320163	4.945083694	0.000176522	0.042549379	1.021365332	UP

IGHG1	4.174424041	6.713647397	4.936963366	0.000179331	0.042762363	1.006931295	UP
ST8SIA4	2.710804772	7.572501909	4.933797148	0.000180438	0.042762363	1.001300845	UP
SNX20	2.136271227	4.461935565	4.927700513	0.000182591	0.042911898	0.990455418	UP
MS4A6A	3.205336218	8.216329506	4.918907401	0.000185742	0.043152594	0.974804276	UP
TRIM59	2.216764722	5.545454118	4.916333423	0.000186675	0.043152594	0.970220788	UP
TRAJ46	2.100967682	0.985726641	4.911877284	0.000188302	0.043174755	0.962283609	UP
ANKRD22	3.31280594	2.75419333	4.903973147	0.000191224	0.043218101	0.948198331	UP
SERPINA3	-3.322960073	6.970493695	-4.903081826	0.000191556	0.043218101	0.946609456	Down
SP110	1.406030042	7.322607653	4.889321364	0.000196764	0.043816225	0.922066408	UP
TRAT1	3.140930951	3.917840962	4.887887813	0.000197314	0.043816225	0.919508076	UP
HLA-DMB	2.864141693	6.446501636	4.86489555	0.00020637	0.045469043	0.878438324	UP
TRAJ28	2.400167717	1.251750324	4.858557367	0.00020894	0.045540758	0.867104432	UP
TRAJ15	2.588607125	1.205949697	4.856151742	0.000209925	0.045540758	0.86280132	UP
AP4B1-AS1	1.586499591	3.731997559	4.850538619	0.00021224	0.045574024	0.852757751	UP
CACFD1	-1.187302165	3.955222167	-4.845959881	0.000214148	0.045574024	0.84456193	Down
LINC01094	3.044576833	4.298065967	4.843318004	0.000215257	0.045574024	0.839831784	UP
IGLV2-23	2.825298453	1.144467933	4.840275352	0.000216542	0.045574024	0.834382933	UP
TMEM66	1.5020972	10.15867913	4.834093033	0.000219177	0.045786814	0.823307749	UP
FAP	3.785853943	6.329676488	4.82957614	0.000221122	0.045853626	0.815212893	UP
P2RY13	2.690886324	4.338910337	4.815369959	0.000227359	0.046106584	0.789736211	UP
EVI2A	2.407418105	8.107832096	4.815005398	0.000227521	0.046106584	0.789082079	UP
MAP4K1	2.239526678	3.433428902	4.814801929	0.000227612	0.046106584	0.788716985	UP
FAM26F	2.081629298	3.349755167	4.811961505	0.000228882	0.046106584	0.783619744	UP
CSF2RA	2.391170725	5.591702193	4.805400442	0.000231843	0.046371903	0.771841687	UP
TRBV23-1	2.035428344	0.801870033	4.782789339	0.000242354	0.048045304	0.731208912	UP
STK17B	2.481807695	9.791009889	4.77922552	0.000244055	0.048045304	0.724798641	UP
LOC100128670	2.750632553	2.491000702	4.776591808	0.00024532	0.048045304	0.720060316	UP
KLRG1	2.064493487	4.323879744	4.772717766	0.000247193	0.048045708	0.713088899	UP
IGLV2-8	2.986110815	1.349250014	4.769562225	0.00024873	0.048045708	0.707409025	UP
SKAP1	2.594084392	3.462460147	4.751006018	0.000257966	0.049199955	0.67398286	UP

CNOT6L	1.306737692	10.37460329	4.750555271	0.000258194	0.049199955	0.673170366	UP
SEMA4D	2.430313353	7.404184186	4.743226553	0.000261942	0.049241999	0.659956397	UP
RGS1	4.35448835	9.008750931	4.740229882	0.00026349	0.049241999	0.654551335	UP
NME8	2.472750681	2.379909612	4.739916126	0.000263653	0.049241999	0.653985351	UP
DAPP1	3.091578979	5.783147452	4.729739061	0.000268986	0.049838475	0.635620287	UP
PSTPIP2	2.020750674	6.204320527	4.727110651	0.000270381	0.049838475	0.630875075	UP

Note: DEGs, differentially expressed genes.
