# Supplementary material for: Upregulated Expression of IL2RB Causes Disorder of Immune Microenvironment in Patients with Kawasaki Disease
Source: Biomed Res Int. 2022 Jul 25;2022:2114699. doi: 10.1155/2022/2114699 (PMC9343205; doi:10.1155/2022/2114699)
Supplement: Supplementary Materials — Supplementary Table 1: clinical data on children whose coronary artery tissues were tested in this study. Supplementary Table 2: the DEGs1 from the comparison between the untreated case group and the control group. Supplementary Table 3: the DEGs2 from the comparison between the treated case group and the control group. Supplementary Table 4: immune cell score matrix estimated by CIBERSORT algorithm. Supplementary Table 5: coexpression analysis of DEGs and immune cell populations. Supplementary Table 6: correlation analysis between the screened 15 core genes and CD4+ memory T cells. [file 2114699.f1.zip › Supplementary Table 4 (1).pdf]

S Table 4. Immune cell score matrix estimated by CIBERSORT algorithm.

	GSM1572233	GSM1572234	GSM1572235	GSM1572236	GSM1572237	GSM1572238	GSM1572239	GSM1572240	GSM1572241	GSM1572242	GSM1572243	GSM1572244	GSM1572245	GSM1572246	GSM1572247
B cells naive	0.0992121	0.1286181	0.0211019	0.0882722	0.0447008	0.0700436	0.127966	0.0746416	0	0.124523	0	0.0801072	0	0	0
B cells memory	0	0	0	0	0	0	0	0	0	0	0	0	0.0205882	0.0070474	0
Plasma cells	0.0140002	0	0.0093556	0.00532	0.0004856	0.0064346	0.017747	0	0.0013331	0.0254329	0	0	0	0.0026464	0
T cells CD8	0	0	0	0.0156397	0	0	0	0	0	0	0	0	0	0	0
T cells CD4 naive	0	0.2933381	0.0216773	0	0.3375311	0.0399107	0.0110456	0.043105	0.0544036	0	0.0436563	0	0.0290908	0.084711	0.1263737
T cells CD4 memory resting	0.2159436	0.2494999	0.2883512	0.1973795	0.2769454	0.2915057	0.2389053	0.3031366	0.2676737	0.3843161	0.4098872	0.4068143	0.3137933	0.2736033	0.3720129
T cells CD4 memory activated	0	0.0223788	0	0	0.0110652	0.0184732	0.0138225	0	0	0	0	0	0	0	0
T cells follicular helper	0	0	0	0.0744706	0	0	0	0	0	0.0222462	0	0	0	0	0
T cells regulatory (Tregs)	0	0	0	0	0	0	0	0	0	0	0	0	0	0	0
T cells gamma delta	0	0	0	0	0	0	0	0	0	0	0	0	0	0	0
NK cells resting	0.0038238	0.026511	0.0385127	0	0.0376967	0.1069223	0.079557	0.0493243	0.0595771	0.0542409	0.0072083	0.043933	0.063603	0.0518049	0.0678795
NK cells activated	0.0198761	0	0	0.0300093	0	0	0	0	0	0.0187906	0.0419664	0.0336471	0.0023484	0.0200104	0.0154374
Monocytes	0.14111	0.042385	0.103938	0.0893479	0.0016229	0.1187477	0.154595	0.068507	0.1125122	0.0545152	0.0829374	0.0382726	0.0651927	0.0356726	0.0362549
Macrophages M0	0	0	0	0	0	0.0237946	0	0.0708368	0	0.0556831	0.0621545	0	0	0.2951772	0
Macrophages M1	0.1040929	0.027311	0.0126819	0.1190918	0	0.0321634	0.0370869	0.1078053	0.0308849	0.1097813	0.082416	0	0.1622646	0	0
Macrophages M2	0.3466569	0.1717662	0.3990136	0.2875987	0.1727613	0.096591	0.2733289	0.1949909	0.444181	0	0.2083477	0.2032678	0.1627547	0.1154667	0.3044167
Dendritic cells resting	0	0	0	0	0	0	0	0	0	0	0	0	0	0	0
Dendritic cells activated	0	0.0173666	0.0118693	0	0.0268012	0	0	0	0	0	0	0.0764498	0.159197	0.0794118	0
Mast cells resting	0.0552845	0.0154756	0.0801444	0.0892297	0.012269	0.0042679	0.0291133	0.0153694	0	0.0938716	0.0614263	0.0201593	0.0121277	0.0270585	0.0571936
Mast cells activated	0	0	0	0	0	0	0	0	0	0	0	0	0	0	0
Eosinophils	0	0	0.013354	0.0036405	0	0.1760766	0.0018862	0.0160691	0.0206067	0	0	0.0973489	0	0	0.0195514
Neutrophils	0	0.0053497	0	0	0.0781209	0.0150687	0.0149464	0.0562141	0.0088277	0.0565991	0	0	0.0090395	0.0073899	0.0008798
