# Supplementary material for: Upregulated Expression of IL2RB Causes Disorder of Immune Microenvironment in Patients with Kawasaki Disease
Source: Biomed Res Int. 2022 Jul 25;2022:2114699. doi: 10.1155/2022/2114699 (PMC9343205; doi:10.1155/2022/2114699)
Supplement: Supplementary Materials — Supplementary Table 1: clinical data on children whose coronary artery tissues were tested in this study. Supplementary Table 2: the DEGs1 from the comparison between the untreated case group and the control group. Supplementary Table 3: the DEGs2 from the comparison between the treated case group and the control group. Supplementary Table 4: immune cell score matrix estimated by CIBERSORT algorithm. Supplementary Table 5: coexpression analysis of DEGs and immune cell populations. Supplementary Table 6: correlation analysis between the screened 15 core genes and CD4+ memory T cells. [file 2114699.f1.zip › Supplementary Table 5 (1).pdf]

S Table 5. Co-expression analysis of DEGs and immune cell populations.

	B cells naive	B cells memory	Plasma cells	T cells CD8	T cells CD4 naive	T cells CD4 memory resting	T cells CD4 memory activated	T cells follicular helper	NK cells resting	NK cells activated	Monocytes	Macrophages M0	Macrophages M1	Macrophages M2	Dendritic cells activated	Mast cells resting	Eosinophils	Neutrophils
RPS26P56	-0.69418	0.05174	-0.37305	-0.4414	-0.01574	0.652256	-0.30689	-0.44756	0.39967	0.154569	-0.3025	0.389175	-0.38998	-0.07823	0.123486	-0.28302	0.202975	0.085292
TRMT1	-0.67215	0.338276	-0.48888	-0.32211	0.222319	0.697464	-0.28057	-0.31069	0.198754	0.232032	-0.606	0.245641	-0.19214	-0.23153	0.415509	-0.23861	-0.05841	0.133966
MTCP1	-0.66954	0.137546	-0.15022	-0.34844	-0.25489	0.586074	-0.4809	-0.32189	0.391059	0.071454	-0.07139	0.190758	-0.15229	0.095926	0.119939	-0.11871	0.042545	0.109147
LETM1	-0.65992	0.442594	-0.36817	-0.30251	-0.19211	0.661015	-0.53245	-0.2733	0.233126	0.229332	-0.28511	0.137581	0.09185	-0.02779	0.443085	-0.21529	-0.0117	-0.078
TTLL12	-0.65038	0.299367	-0.57274	-0.32851	0.152809	0.594104	-0.17127	-0.32931	0.334016	0.086382	-0.48634	0.407642	-0.1552	-0.27434	0.31373	-0.40025	0.118229	0.065537
TRMT61A	-0.64317	0.399598	-0.53165	-0.20409	0.084908	0.73993	-0.29613	-0.18618	0.171996	0.339725	-0.57408	0.232583	-0.05531	-0.30928	0.477019	-0.20795	0.103713	-0.01584
MID1IP1-AS1	-0.62782	0.446689	-0.41443	-0.37799	-0.17831	0.673931	-0.52951	-0.38924	0.164105	0.22021	-0.34459	0.21738	-0.08746	0.041932	0.556181	-0.17036	-0.102	-0.20543
PFN1P3	-0.62728	0.408309	-0.26314	-0.05493	-0.13211	0.563793	-0.42799	-0.03143	0.360137	0.232567	-0.41624	0.292226	-0.11607	-0.21888	0.478862	-0.08763	0.088163	0.104683
MTA1	-0.61665	0.115414	-0.37829	-0.20896	-0.03208	0.847417	-0.46493	-0.17256	0.102324	0.450019	-0.48751	0.169563	-0.13738	-0.12988	0.177276	0.074847	-0.00256	-0.02179
HOOK2	-0.61538	0.33721	-0.24748	-0.28481	-0.38275	0.556706	-0.46228	-0.28812	0.477763	0.091038	-0.01887	0.147081	0.038602	0.003556	0.31733	-0.29338	0.222032	0.023618
RPL7P18	-0.61475	0.199806	-0.46209	0.070311	-0.13649	0.463762	-0.36949	0.039792	0.227387	0.304209	-0.25137	0.232248	-0.0065	-0.09194	0.212246	-0.24833	0.181443	0.062826
MLX	-0.61402	0.246098	-0.47216	-0.41809	0.118507	0.602575	-0.18841	-0.42065	0.552239	-0.03027	-0.46892	0.297056	-0.30831	-0.22054	0.319228	-0.47516	0.252808	0.203974
LOC100289473	-0.60217	0.243625	-0.44044	-0.41372	0.086389	0.563515	-0.11367	-0.46341	0.539068	-0.04712	-0.24386	-0.05298	-0.28258	0.035156	0.26199	-0.42798	0.209346	-0.00591
AURKC	-0.5904	0.190727	-0.2896	-0.49109	-0.09768	0.688041	-0.17726	-0.47827	0.663233	-0.03008	-0.28232	0.203658	-0.38986	-0.18617	0.29725	-0.32659	0.506695	0.045092
COASY	-0.57477	0.305056	-0.51197	-0.29216	0.045341	0.657492	-0.15091	-0.27702	0.438427	0.046917	-0.43565	0.264733	-0.13132	-0.28271	0.343447	-0.38332	0.234633	0.056282
HMGB1P24	-0.57405	0.254425	-0.43285	-0.50697	0.077109	0.561451	-0.05567	-0.5359	0.524131	-0.096	-0.20459	0.120713	-0.20402	-0.10647	0.268451	-0.55993	0.203065	0.14255
POR	-0.56958	0.618643	-0.38578	-0.20466	-0.16563	0.554479	-0.34441	-0.20087	0.290102	0.25864	-0.26794	0.145583	0.242167	-0.26631	0.588224	-0.31213	0.16442	-0.07542
LONP1	-0.56565	0.185451	-0.49826	-0.33225	-0.0059	0.761606	-0.25816	-0.32492	0.336854	0.291617	-0.43376	0.204535	-0.15788	-0.23756	0.248897	-0.27069	0.327046	-0.07426
C3orf27	-0.56313	0.251374	-0.1665	-0.39526	-0.09479	0.680079	-0.23191	-0.35557	0.3468	0.298542	-0.27919	0.28832	-0.32804	-0.22606	0.401138	-0.18011	0.252097	-0.05877
TOMM40	-0.55927	0.437247	-0.36	-0.2184	-0.30065	0.727183	-0.46952	-0.21972	0.282031	0.392199	-0.2766	0.219054	0.048871	-0.1566	0.479707	-0.11978	0.114244	-0.22936
TPM3P7	-0.5394	0.205705	-0.3737	0.003013	0.030643	0.53539	-0.37983	0.058405	0.205069	0.268444	-0.52544	0.321642	0.042272	-0.35968	0.218659	-0.16028	0.148837	0.33524
JMJD4	-0.53394	0.26527	-0.21388	-0.35668	0.011917	0.774478	-0.29642	-0.29564	0.388674	0.155449	-0.49803	0.250193	-0.27502	-0.24686	0.350933	-0.01938	-0.01385	0.08489
IL20RB	-0.53012	0.254797	-0.30777	-0.56542	-0.03978	0.781698	-0.17261	-0.5432	0.593183	-0.02825	-0.34775	0.180842	-0.27569	-0.24341	0.336394	-0.31091	0.2831	0.11755
CLUH	-0.52775	0.26191	-0.39759	-0.23511	-0.17375	0.813041	-0.54679	-0.20931	0.146352	0.389984	-0.44146	0.124744	-0.00236	-0.09529	0.341071	-0.02464	0.010256	-0.09466
OSGIN1	-0.52452	0.32395	-0.21536	-0.13027	-0.31283	0.436505	-0.74758	-0.10021	-0.24501	0.582538	-0.20269	0.419745	0.195832	0.037245	0.345631	0.068132	-0.26083	-0.24893
PKDCC	-0.51627	0.331111	-0.01002	-0.06734	-0.24139	0.336464	-0.44746	-0.00117	0.050287	0.296861	-0.00032	0.114033	0.119269	0.154982	0.190479	0.185112	-0.24917	-0.41549
MIR1276	-0.51454	0.152497	-0.31901	-0.26099	-0.24534	0.752661	-0.56278	-0.23138	0.068951	0.50795	-0.3515	0.235858	-0.2032	0.004227	0.342125	-0.04482	0.084907	-0.26927
CACFD1	-0.51276	0.325	-0.21632	-0.1913	-0.08726	0.575805	-0.47709	-0.09533	0.027498	0.34113	-0.45039	0.440614	0.035179	-0.21966	0.30424	0.159601	-0.12893	-0.21797
MAPK12	-0.51245	0.23328	-0.32849	-0.33666	-0.10395	0.609962	-0.35544	-0.33171	0.131351	0.446751	-0.28008	0.252887	-0.11195	-0.17898	0.379546	-0.32391	0.232484	-0.02371
AURKAIP1	-0.5108	0.243765	-0.12514	-0.16629	-0.34278	0.743004	-0.56686	-0.07995	0.348275	0.238118	-0.22862	0.026021	0.167757	-0.12789	0.178215	0.093069	0.125224	0.075573
THOC6	-0.51026	0.31523	-0.23109	-0.03655	-0.30495	0.624164	-0.40452	-0.01642	0.452968	0.174612	-0.25393	0.121082	0.000922	-0.19582	0.35684	-0.08032	0.254601	0.115633
KCNG2	-0.5057	0.060338	-0.44285	-0.36891	-0.006	0.562138	-0.22445	-0.42063	0.425716	0.173914	-0.30695	0.315518	-0.3889	-0.069	0.185414	-0.42358	0.225635	0.030567
TOR4A	-0.50252	0.2203	-0.06307	-0.29778	-0.28549	0.666897	-0.38644	-0.21238	0.440261	0.208352	-0.22854	0.545085	-0.05193	-0.3337	0.157317	0.061928	0.071678	0.022647
SLC27A5	-0.49494	0.233394	-0.0324	-0.49426	-0.12534	0.636556	-0.3594	-0.41453	0.520915	-0.07889	-0.24038	0.241748	-0.13887	-0.14751	0.215996	-0.12561	-0.05901	0.302182
COMTD1	-0.49293	0.286917	-0.24252	-0.06811	-0.17385	0.723555	-0.45466	-0.00181	0.357521	0.249207	-0.4856	0.238996	-0.01675	-0.32761	0.34967	0.003931	0.125043	0.212917
MCAT	-0.48751	0.456794	-0.31908	-0.22187	-0.25118	0.716837	-0.4396	-0.18923	0.344844	0.261742	-0.30924	0.120562	0.163161	-0.21638	0.454091	-0.16471	0.082448	-0.0419
MTHFR	-0.4829	0.071807	-0.21514	-0.32688	-0.10321	0.861775	-0.41318	-0.24015	0.24366	0.339998	-0.41067	0.069712	-0.16826	-0.11992	0.128849	0.098728	0.12142	-0.11084
TBRG4	-0.4762	0.315702	-0.16351	-0.19495	-0.25811	0.609215	-0.45531	-0.14437	0.321944	0.350054	-0.22379	0.217013	0.165605	-0.27179	0.262436	-0.09559	0.169008	0.078797
SNORD96B	-0.46991	0.320768	-0.1634	-0.48787	-0.31358	0.724877	-0.31986	-0.45397	0.528273	0.040086	-0.13473	0.17509	-0.07323	-0.19387	0.392678	-0.2674	0.259334	0.100492
MIR1913	-0.45982	0.124115	-0.44164	-0.33012	-0.06078	0.656576	-0.43011	-0.30666	0.257664	0.218023	-0.47943	0.291313	-0.17726	-0.17563	0.304169	-0.3775	0.223326	0.188794

RSPO4	-0.45852	0.278456	-0.17794	0.106456	-0.21017	0.164688	-0.51568	0.104697	-0.5215	0.497849	0.050865	0.020118	0.308074	0.307473	0.213266	0.229229	-0.36184	-0.48888
MIR635	-0.45843	0.303484	-0.0775	-0.25303	-0.24638	0.636987	-0.54816	-0.1952	0.264245	0.308203	-0.28918	0.13291	0.034058	-0.18539	0.382852	-0.07193	0.077234	0.239124
IL1RL1	-0.45612	0.641649	-0.28466	-0.30793	-0.04052	0.475216	-0.20791	-0.28642	0.231937	0.309013	-0.40133	0.58366	0.017121	-0.47965	0.706346	-0.29474	0.002409	-0.11716
SIAH2-AS1	-0.45265	0.089492	-0.37692	-0.58092	0.233131	0.633808	-0.00061	-0.58528	0.519889	-0.12833	-0.44147	0.157791	-0.48272	-0.20627	0.264054	-0.49686	0.251449	0.325537
TPGS1	-0.44067	0.017618	-0.11205	-0.02432	-0.17351	0.636736	-0.3479	0.068578	0.430021	0.18539	-0.28875	0.126565	-0.01913	-0.2563	-0.02874	0.043052	0.251693	0.27799
NSFP1	-0.43441	0.253228	-0.08139	-0.31196	-0.26061	0.684836	-0.23279	-0.23003	0.451162	0.272951	-0.10101	0.099902	-0.04764	-0.1909	0.236839	-0.11121	0.279052	-0.22206
RPS27P15	-0.433	0.121754	-0.22249	-0.47726	0.074703	0.498846	-0.00208	-0.46249	0.706982	-0.21781	-0.3017	0.243496	-0.35064	-0.3337	0.197421	-0.45639	0.469723	0.430982
GGN	-0.42922	0.242833	-0.43028	-0.44135	0.028717	0.716138	-0.10968	-0.45201	0.465812	0.213915	-0.44618	0.307145	-0.34147	-0.30144	0.39633	-0.37687	0.290727	-0.103
RPL15P18	-0.42795	0.226225	-0.09311	-0.48908	0.047841	0.650122	-0.10506	-0.43732	0.596369	-0.10125	-0.3149	0.081379	-0.21185	-0.30267	0.253295	-0.2222	0.176423	0.390779
LOC101929372	-0.4276	-0.00824	-0.40132	0.010768	-0.00759	0.638548	-0.31934	0.035275	0.249929	0.334217	-0.46609	0.102933	-0.16233	-0.18166	0.102097	-0.16953	0.231977	0.187241
CDC42EP4	-0.41131	0.371057	-0.28389	-0.04121	-0.13886	0.673324	-0.45929	0.050088	-0.02097	0.442755	-0.3977	0.049046	0.300348	-0.23542	0.365784	0.018741	-0.05585	-0.06844
SETP6	-0.41067	0.354919	-0.22909	-0.50209	-0.23137	0.492788	-0.2696	-0.50772	0.342487	0.157196	-0.03159	0.144195	0.036969	-0.13993	0.429976	-0.5187	0.300204	0.038429
HMGB1P19	-0.40839	0.277391	-0.40475	-0.37954	0.171695	0.437851	0.054235	-0.41831	0.50269	-0.03938	-0.37593	0.315596	-0.18387	-0.45218	0.355516	-0.56386	0.362311	0.391302
MCF2L-AS1	-0.40363	0.377493	-0.31563	-0.35463	0.15501	0.485725	-0.30758	-0.3397	0.242193	0.057889	-0.54241	0.272929	-0.10997	-0.24873	0.505099	-0.37369	-0.17377	0.401366
TELO2	-0.39902	0.112545	-0.29948	-0.26203	0.006336	0.784883	-0.36479	-0.19649	0.125197	0.515091	-0.54061	0.192031	-0.14054	-0.31222	0.259184	-0.08563	0.184771	0.027541
NECAB3	-0.39489	0.07235	-0.12828	-0.13947	-0.19419	0.78744	-0.54774	-0.04268	0.173665	0.478544	-0.44792	0.237183	-0.07157	-0.21065	0.135218	0.186255	-0.00903	0.02354
ESRRA	-0.39267	0.212892	-0.20964	-0.16755	-0.15707	0.82266	-0.52787	-0.08383	0.094105	0.464779	-0.47946	0.201109	0.106226	-0.28409	0.24131	0.152173	-0.11055	0.072219
PRSS45	-0.3913	0.349759	-0.13451	-0.34607	-0.21101	0.772811	-0.52305	-0.25988	0.240959	0.187622	-0.3459	0.057292	0.168072	-0.17047	0.346144	0.021945	-0.16048	0.141053
SNORD121A	-0.38641	0.282974	-0.07778	-0.49223	-0.23202	0.556247	-0.20949	-0.46989	0.621255	-0.10142	-0.04734	0.142778	-0.16144	-0.0719	0.305236	-0.31513	0.091	0.083989
S1PR2	-0.38112	0.518402	-0.03029	0.014131	-0.40968	0.451066	-0.47304	0.104524	0.089604	0.480358	-0.11961	0.300543	0.296464	-0.24334	0.493888	0.050344	-0.00645	-0.28971
ABCB6	-0.36151	0.187157	-0.1361	-0.75139	0.030937	0.516384	0.11789	-0.72806	0.737457	-0.259	-0.09518	0.157506	-0.3365	-0.26175	0.253973	-0.60489	0.458329	0.22827
CDS1	-0.35656	0.307266	0.013128	-0.65465	-0.18658	0.64129	-0.37152	-0.57487	0.420187	0.074832	-0.30419	0.477966	-0.22345	-0.30063	0.449027	-0.18708	0.037693	0.17281
ST6GALNAC4P1	-0.35032	0.01633	-0.20167	-0.4035	-0.04191	0.875707	-0.37231	-0.3375	0.192898	0.340148	-0.47658	0.134615	-0.20931	-0.25762	0.201916	-0.06349	0.079956	0.273474
GPR56	-0.34988	0.305912	-0.33568	-0.10025	-0.12641	0.522545	-0.43036	-0.06526	-0.06044	0.530739	-0.37842	0.409075	0.11118	-0.25686	0.409358	-0.20474	-0.10436	-0.0571
TWIST2	-0.34204	0.361794	-0.40237	-0.19954	0.118913	0.368787	-0.03865	-0.20077	0.456756	0.03019	-0.37506	0.134017	-0.15641	-0.1989	0.435293	-0.53661	0.168866	-0.01458
TMED1	-0.32672	0.143564	0.02088	-0.09291	-0.29142	0.644245	-0.33571	0.004167	0.505096	0.262628	-0.20173	0.159374	-0.0066	-0.28417	0.109081	0.047178	0.187555	0.099461
MIR601	-0.32505	0.040656	-0.15228	-0.37665	0.121742	0.584544	-0.1676	-0.33623	0.369897	0.136354	-0.42877	0.076887	-0.46486	-0.13521	0.268984	-0.28531	0.139009	0.243595
SERPINA3	-0.32393	0.424578	0.00286	0.040426	-0.31251	0.449317	-0.49056	0.176036	-0.0066	0.388688	-0.16706	0.198735	0.438335	-0.24601	0.332822	0.105504	-0.16386	-0.05511
RNY4P6	-0.31226	0.300557	0.00917	-0.23273	-0.17799	0.597774	-0.18965	-0.12814	0.649316	-0.06891	-0.29151	0.109984	-0.07676	-0.38936	0.356948	-0.16676	0.299581	0.345529
ASB6	-0.30776	0.238542	-0.22613	-0.19161	-0.15276	0.590227	-0.15831	-0.18988	0.441559	0.36848	-0.29808	0.253259	-0.20685	-0.3626	0.399888	-0.29462	0.393395	-0.01457
EIF4A1P10	-0.28809	0.330532	-0.09443	-0.35097	-0.38996	0.498092	-0.18345	-0.31812	0.639775	0.116651	-0.03784	0.377622	-0.11253	-0.29287	0.410803	-0.40112	0.414538	-0.11596
TRHR	-0.24922	0.159793	-0.09361	-0.18828	-0.29677	0.61277	-0.11705	-0.12843	0.42715	0.248356	-0.06339	0.149466	-0.10879	-0.21831	0.241398	-0.19886	0.268	-0.15598
RNU1-19P	-0.17961	0.322796	0.105674	-0.37302	-0.31924	0.529815	-0.12392	-0.30152	0.532496	0.081069	0.054018	0.216535	0.141392	-0.34133	0.246653	-0.19986	0.048953	0.11413
RPS2P7	-0.14477	0.072881	0.185573	-0.31921	-0.27048	0.805324	-0.28099	-0.14581	0.265695	0.368709	-0.15933	0.049277	0.105364	-0.37436	0.095153	0.080038	0.084241	0.16445
TRAJ28	0.044352	-0.10063	-0.10988	0.074634	0.30037	-0.41328	0.455098	-0.00873	0.318947	-0.70246	0.125233	-0.29392	-0.11848	0.088787	-0.16327	-0.35753	0.262651	0.45357
TRAV21	0.100857	-0.13391	-0.00992	-0.1941	0.332129	-0.24549	0.402551	-0.24642	0.389518	-0.49947	-0.05327	0.201243	-0.28135	-0.22837	-0.11845	-0.42189	0.148871	0.642742
XAF1	0.125987	-0.16656	0.060698	0.329175	-0.05942	-0.62057	0.019404	0.202607	-0.46547	-0.19125	0.422726	-0.17635	0.081712	0.542242	-0.22203	0.248705	-0.28517	-0.18293
CCDC11P1	0.133705	-0.17111	0.296782	0.410655	-0.31688	-0.49502	-0.28412	0.362943	-0.5598	0.140049	0.449652	-0.22498	0.219714	0.527205	-0.23186	0.553871	-0.1859	-0.34037
LOC101929241	0.139603	-0.24365	-0.08373	-0.22061	0.485605	-0.17711	0.499993	-0.28007	0.208175	-0.49985	-0.08319	-0.02295	-0.26338	-0.15486	-0.22911	-0.36021	0.059688	0.648521
RALYL	0.16067	0.206243	0.051108	0.161018	0.15686	-0.45062	0.137605	0.135714	-0.42376	-0.13474	0.019844	0.025114	0.111241	0.12368	0.177732	0.24665	-0.33651	-0.36285
DSPP	0.166408	-0.03616	0.005725	0.418709	-0.26062	-0.52255	0.024189	0.28275	-0.30622	-0.0128	0.393978	-0.08589	0.134174	0.322666	-0.05951	0.259263	0.110648	-0.44846
P2RX7	0.173612	-0.2875	-0.08108	0.206692	0.248075	-0.72474	0.368642	0.062614	-0.09771	-0.43836	0.259743	-0.12928	-0.17007	0.314114	-0.32838	-0.12474	0.184054	0.024068
TRBV23-1	0.182644	-0.27183	-0.00365	0.196825	0.322145	-0.19041	0.434843	0.175237	0.352834	-0.32501	-0.09022	-0.1744	-0.13376	-0.25202	-0.3169	-0.18456	0.365454	0.584962
HLA-DPB1	0.183749	-0.13383	-0.00666	0.360983	-0.04474	-0.72756	0.387301	0.194026	0.01332	-0.32554	0.484305	-0.10285	-0.08452	0.289361	-0.18725	-0.07687	0.306255	-0.24166
PARP14	0.196458	0.015746	0.045565	0.1892	0.045201	-0.8093	0.318743	0.063401	-0.19206	-0.40814	0.436725	0.016566	0.05295	0.275921	-0.04767	-0.12686	0.001531	-0.1498

KLRG1	0.201725	-0.24422	0.090814	-0.0278	0.546561	-0.42453	0.535977	-0.05184	0.116966	-0.60166	0.060186	-0.30465	-0.04987	-0.00569	-0.4037	-0.17523	-0.07726	0.618198
FILIP1L	0.20976	-0.08014	-0.00446	0.252342	0.099927	-0.56046	0.317266	0.147389	-0.37565	-0.14158	0.313411	-0.1822	-0.10964	0.418756	-0.06469	0.126101	-0.14341	-0.55522
SLFN12L	0.210682	-0.01737	-0.13452	0.345815	0.30127	-0.76552	0.593595	0.1895	-0.01898	-0.52405	0.288104	-0.23501	0.0063	0.177976	-0.11494	-0.23787	0.100153	0.012986
MX1	0.219545	-0.25709	0.177259	0.50953	-0.1581	-0.60834	0.074378	0.437106	-0.35747	-0.15114	0.461687	-0.28209	0.149381	0.441886	-0.3703	0.414816	-0.00999	-0.28026
UBASH3A	0.223321	-0.5459	-0.19381	-0.01644	0.530398	-0.41031	0.671894	-0.15734	0.043006	-0.39851	0.124525	-0.26415	-0.42643	0.178105	-0.53093	-0.30421	0.268892	0.19466
IFIH1	0.233684	0.029049	0.225546	0.443589	-0.05848	-0.72401	0.11342	0.401179	-0.24646	-0.39634	0.37278	-0.20657	0.342315	0.247268	-0.1455	0.250647	-0.14741	0.034565
OAS1	0.233837	-0.09723	0.184197	0.436335	-0.06206	-0.80392	0.120149	0.356651	-0.37474	-0.29755	0.479158	-0.2013	0.215898	0.444374	-0.2365	0.236233	-0.17041	-0.19988
TRAV29DV5	0.235765	-0.26586	0.069932	-0.20471	0.34744	-0.27363	0.557511	-0.25988	0.343623	-0.66013	0.109009	-0.18451	-0.29485	-0.02699	-0.26527	-0.295	0.181752	0.516511
MS4A6A	0.239568	-0.25722	-0.02516	0.241112	0.212028	-0.85305	0.357439	0.075708	-0.23355	-0.43366	0.44666	-0.22363	-0.04563	0.486659	-0.33138	-0.18754	-0.10091	0.026479
TRAV12-1	0.243112	-0.25806	-0.03992	-0.19871	0.527138	-0.26327	0.682802	-0.25226	0.336067	-0.64076	-0.04013	-0.18369	-0.31958	-0.165	-0.23675	-0.40615	0.269901	0.582006
EPS8L3	0.258448	-0.4072	0.130942	0.36607	0.214601	-0.71002	0.128979	0.318634	-0.38962	-0.20982	0.231567	-0.22641	-0.04939	0.484214	-0.50822	0.238393	-0.20587	-0.11719
C4orf50	0.271948	-0.20174	0.125821	0.311742	-0.04422	-0.49952	0.102905	0.245131	-0.63342	0.249461	0.367196	-0.06949	0.200252	0.244545	-0.26693	0.329136	-0.10641	-0.44465
BTN3A1	0.281467	-0.30912	0.143123	0.145839	0.396993	-0.81106	0.469679	0.07482	-0.09608	-0.60885	0.266754	-0.12502	-0.1345	0.267128	-0.42186	-0.09292	-0.1306	0.275694
DCDC2C	0.285074	-0.2451	-0.06335	0.451892	0.130655	-0.46902	0.063163	0.375874	-0.70094	0.19128	0.112997	-0.13063	0.182278	0.302973	-0.31106	0.384186	-0.32621	-0.38504
ITGAL	0.288088	-0.25699	-0.05836	0.150307	0.429585	-0.83411	0.549679	0.010099	-0.0886	-0.53042	0.245875	-0.13666	-0.19221	0.261101	-0.28193	-0.34141	0.027066	0.18568
CLEC10A	0.289879	-0.21354	-0.20421	0.501997	0.198143	-0.75326	0.335575	0.326576	-0.30518	-0.30755	0.308041	-0.36089	0.151463	0.399713	-0.31273	-0.09618	-0.06204	-0.03688
MX2	0.291382	-0.27293	0.191348	0.390282	0.011768	-0.70276	0.281716	0.308604	-0.22654	-0.36064	0.451361	-0.36542	-0.01918	0.433499	-0.31252	0.163768	0.05371	-0.09997
SP110	0.292045	-0.21526	-0.00723	0.167628	0.399543	-0.65059	0.491079	0.050971	-0.27752	-0.46812	0.246381	-0.42853	-0.01562	0.307056	-0.26901	-0.06338	-0.11425	0.152501
LPXN	0.29313	-0.31103	0.047793	0.068439	0.240562	-0.70773	0.533563	-0.04796	0.15274	-0.55195	0.34352	-0.13599	-0.20888	0.177876	-0.35822	-0.29826	0.323416	0.16834
ZDHHC4P1	0.294274	-0.12955	0.05805	0.206517	0.050237	-0.71403	0.357928	0.084378	-0.39877	-0.22968	0.557658	-0.29016	0.1962	0.382815	-0.23813	-0.02257	-0.04379	-0.26795
HLA-DRA	0.295515	-0.23746	0.068966	0.31691	-0.05928	-0.76238	0.334219	0.180125	0.042786	-0.36664	0.444763	0.021057	-0.11445	0.242172	-0.26007	-0.12217	0.282527	-0.07897
OAS2	0.300711	-0.11741	0.194113	0.508375	-0.12172	-0.68162	0.077362	0.444947	-0.39521	-0.24803	0.426038	-0.26178	0.303994	0.345624	-0.21542	0.271596	-0.16781	-0.01804
NAGK	0.304893	-0.45522	0.0299	0.061717	0.416838	-0.657	0.444303	-0.06021	-0.35145	-0.2906	0.242039	-0.28172	-0.35831	0.458493	-0.38926	-0.05169	-0.08014	-0.06655
LINC01094	0.305193	-0.18607	-0.01372	0.191313	0.090835	-0.81866	0.391087	0.024365	-0.13715	-0.45394	0.554562	-0.27965	0.062974	0.441942	-0.26989	-0.29458	0.013206	-0.0096
MAP4K1	0.311051	-0.21773	-0.05755	0.049136	0.46321	-0.78812	0.57838	-0.08466	-0.15596	-0.51204	0.258188	-0.15558	-0.08948	0.218156	-0.28799	-0.32396	-0.04489	0.164176
SKAP1	0.313239	-0.32575	-0.18868	0.085793	0.650737	-0.49569	0.783993	-0.01172	0.004921	-0.50063	-0.05325	-0.16822	-0.28871	-0.07671	-0.30412	-0.32877	0.187924	0.317911
CXCR2	0.313992	-0.14377	0.152767	-0.07466	0.503274	-0.42715	0.614704	-0.05398	0.31141	-0.67463	-0.00516	-0.00484	-0.00833	-0.29132	-0.32226	-0.27191	-0.03668	0.686562
FLT3	0.317264	-0.40287	-0.21124	0.019287	0.514256	-0.42066	0.791692	-0.1169	0.23963	-0.39283	0.046594	-0.12443	-0.45426	-0.05371	-0.32795	-0.49978	0.420005	0.209278
CIITA	0.317606	-0.01541	-0.00407	0.22436	0.126882	-0.81484	0.468104	0.054326	-0.20096	-0.3488	0.459067	-0.14742	0.049223	0.296707	-0.09022	-0.13924	-0.02626	-0.2483
PRND	0.323543	-0.36955	0.35062	0.461834	-0.26889	-0.73332	-0.00791	0.389147	-0.3724	-0.11814	0.706103	-0.34928	0.209691	0.607771	-0.50315	0.295131	-0.13146	-0.15389
LOC101927156	0.324151	0.014281	0.031171	0.100343	0.421714	-0.79435	0.627714	0.005929	0.106368	-0.68766	0.17923	-0.05244	-0.04057	-0.0015	-0.06631	-0.38208	-0.02568	0.33435
RSAD2	0.326563	-0.08767	0.445052	0.512239	-0.37192	-0.59809	-0.04231	0.510812	-0.25753	-0.14817	0.525962	-0.13886	0.381517	0.237815	-0.25886	0.510628	-0.09667	-0.13259
DOCK10	0.332054	-0.32829	0.033536	0.022248	0.28252	-0.68008	0.552641	-0.09124	-0.08308	-0.48111	0.330471	-0.09277	-0.21557	0.189967	-0.30478	-0.27066	0.165727	0.139293
HLA-DPA1	0.332197	-0.19569	0.159915	0.352206	-0.11835	-0.79208	0.296081	0.239049	-0.11848	-0.30556	0.529425	0.009626	0.061811	0.23957	-0.27536	0.012994	0.156953	-0.12936
LOC101927095	0.333245	-0.21584	0.28491	-0.2125	0.121344	-0.44806	0.392421	-0.26977	0.21542	-0.40888	0.35945	-0.04188	-0.11013	0.003221	-0.25694	-0.28612	0.117067	0.40489
SIRPG	0.334162	-0.11244	0.003253	-0.04566	0.59836	-0.35754	0.709112	-0.07609	0.335289	-0.67284	-0.11342	-0.22316	-0.18075	-0.21558	-0.15097	-0.38376	0.030718	0.623231
TAGAP	0.334187	-0.37164	0.008765	0.326013	0.249917	-0.74658	0.54158	0.195218	-0.04388	-0.38469	0.288808	-0.09018	-0.22418	0.170211	-0.3741	-0.13834	0.239889	0.084404
TRAJ46	0.337804	-0.02983	-0.10735	0.151204	0.513658	-0.45784	0.782451	0.07347	0.230681	-0.5923	-0.01775	-0.29578	-0.06873	-0.20954	-0.06655	-0.36278	0.228592	0.377189
ARHGEF6	0.340086	-0.40456	-0.04658	0.266486	0.417804	-0.71109	0.440834	0.147376	-0.29619	-0.47089	0.209648	-0.35005	-0.10342	0.414225	-0.47297	-0.00679	-0.19949	0.115785
LCP2	0.340446	-0.26547	-0.04631	0.067499	0.35418	-0.7609	0.633899	-0.08003	-0.07667	-0.50135	0.358901	-0.18656	-0.16104	0.23342	-0.28519	-0.36406	0.127117	0.085422
SNX20	0.341015	-0.19411	-0.05303	0.101851	0.381544	-0.76591	0.636599	-0.02214	-0.01318	-0.60437	0.337919	-0.3524	-0.02339	0.230039	-0.28513	-0.37202	0.15068	0.13189
TRAJ21	0.343925	-0.1326	-0.07186	-0.05333	0.570374	-0.63311	0.705553	-0.14676	0.040314	-0.61701	0.047063	-0.1167	-0.13912	-0.07412	-0.15437	-0.41148	0.115484	0.365442
TLR6	0.344005	-0.23301	0.076193	0.082631	0.34682	-0.80222	0.520783	-0.02662	-0.1565	-0.4968	0.324234	-0.03419	-0.06092	0.175034	-0.30711	-0.23355	-0.1006	0.227191
FCGR3B	0.348272	-0.26385	0.035791	-0.05926	0.558472	-0.42248	0.543243	-0.08636	0.092668	-0.50736	-0.05084	-0.12776	-0.07438	-0.16564	-0.313	-0.36065	-0.03146	0.728104
FCGR2C	0.352392	-0.29532	-0.00224	0.228076	0.146207	-0.83133	0.398117	0.080064	-0.16589	-0.49676	0.513349	-0.32456	-0.00292	0.510301	-0.36569	-0.25475	-0.05295	0.005332
CLDN1	0.353803	-0.1249	0.155452	0.439578	0.104419	-0.57591	0.180504	0.420046	-0.23271	-0.31118	0.042459	-0.16936	-0.06915	0.210157	-0.06815	0.259635	-0.09261	-0.07119

PARP9	0.35855	-0.11011	0.259464	0.426959	-0.15298	-0.73547	0.121195	0.384562	-0.38397	-0.24027	0.500637	-0.09897	0.384726	0.234442	-0.25663	0.20929	-0.14894	-0.01331
AQP9	0.36253	-0.22372	0.113172	-0.22738	0.204797	-0.49844	0.609341	-0.28189	0.33692	-0.57052	0.36104	0.01576	-0.08069	-0.10721	-0.29433	-0.56498	0.314526	0.397502
P2RY13	0.362606	-0.33767	0.22649	0.181249	0.16153	-0.60385	0.284692	0.11487	-0.30391	-0.32465	0.339584	-0.21849	0.005399	0.259751	-0.35513	0.054855	-0.18266	0.308551
GPR171	0.362945	-0.19357	-0.14878	-0.00351	0.361942	-0.34837	0.724994	-0.10089	0.269112	-0.4249	0.121943	-0.24467	-0.15641	-0.15951	-0.12919	-0.62417	0.423875	0.375364
SAMD9	0.363171	-0.21411	0.198534	0.191836	0.195686	-0.764	0.47178	0.119532	-0.13817	-0.52155	0.374039	-0.10005	-0.02847	0.195914	-0.29449	-0.00819	-0.0594	0.111289
CSF2RA	0.364001	-0.18793	-0.08204	0.158281	0.284026	-0.70272	0.702299	-0.00301	0.142289	-0.43048	0.294714	-0.13698	-0.25524	0.082075	-0.14757	-0.43187	0.379882	-0.00403
NME8	0.364063	-0.48638	0.160755	0.087583	0.175361	-0.53547	0.516218	-0.02157	0.142268	-0.39264	0.334674	-0.13729	-0.48201	0.254331	-0.39211	-0.16279	0.198003	0.124428
TRAJ9	0.364904	-0.383	-0.09143	-0.00135	0.507948	-0.18346	0.724186	-0.05566	0.106198	-0.39438	-0.07256	-0.24736	-0.27203	-0.20922	-0.30921	-0.25201	0.279612	0.4473
IFIT2	0.365402	-0.27476	0.38763	0.45189	-0.09115	-0.69758	0.1279	0.432025	-0.33879	-0.24965	0.453575	-0.26045	0.160054	0.342449	-0.37983	0.402106	-0.10899	-0.02944
CXorf21	0.365621	-0.2822	0.02357	0.12057	0.341006	-0.83143	0.680821	-0.02045	0.005688	-0.5519	0.435107	-0.25595	-0.20991	0.326474	-0.33858	-0.33231	0.105004	-0.02955
ITK	0.367056	-0.22051	-0.14559	0.02041	0.572868	-0.64181	0.770749	-0.10438	0.073224	-0.52109	0.048048	-0.10112	-0.2854	-0.04687	-0.17631	-0.48713	0.235516	0.252723
XCL1	0.367349	-0.06355	0.116381	0.405528	-0.0728	-0.74771	0.351074	0.300564	0.062264	-0.45765	0.541572	-0.3608	0.319292	0.210753	-0.23455	-0.1833	0.158973	0.144493
AOAH	0.368172	-0.38879	-0.02758	0.201199	0.24788	-0.77026	0.498972	0.0427	-0.11797	-0.42812	0.394281	-0.27038	-0.15439	0.367005	-0.41257	-0.21971	0.136733	0.032438
STK17B	0.369877	-0.34214	0.02482	0.042531	0.461172	-0.7049	0.669069	-0.05037	0.066617	-0.53832	0.17475	-0.05861	-0.33461	0.069817	-0.29817	-0.41181	0.175904	0.308389
TLR7	0.37581	-0.37121	0.288666	0.226947	-0.02564	-0.81952	0.331504	0.148001	-0.14379	-0.41942	0.623352	-0.21315	-0.02949	0.447076	-0.46582	0.017097	0.054031	-0.07842
CCR5	0.378275	-0.23763	0.078581	0.235414	-0.00076	-0.81092	0.508671	0.092606	-0.02435	-0.39907	0.593366	-0.16034	-0.07043	0.328656	-0.2857	-0.21169	0.25477	-0.23132
PSTPIP2	0.380538	-0.16685	0.001628	-0.09621	0.363594	-0.65101	0.595668	-0.18513	0.023267	-0.56509	0.29625	-0.17776	0.048463	0.038777	-0.2499	-0.48568	0.114279	0.34075
HLA-DMA	0.382246	-0.35947	0.140224	0.309414	0.006973	-0.80319	0.387756	0.191589	0.004128	-0.37268	0.482524	-0.12537	-0.13985	0.325099	-0.40146	-0.08219	0.267154	-0.11128
MS4A4E	0.384583	-0.2463	0.002563	0.268073	0.459463	-0.73675	0.498594	0.137812	-0.36538	-0.30938	0.192419	-0.33158	-0.03422	0.277052	-0.29718	-0.02847	-0.23477	0.091395
CAMK4	0.385894	-0.41453	-0.09156	-0.04328	0.561073	-0.38167	0.754532	-0.13105	0.130844	-0.42918	-0.01755	-0.21507	-0.39186	-0.1103	-0.30476	-0.41943	0.357691	0.413199
PLXNC1	0.387749	-0.35735	0.143978	-0.04091	0.303369	-0.63702	0.489384	-0.12895	-0.13599	-0.44374	0.289603	-0.00153	-0.17603	0.130518	-0.36022	-0.16859	-0.05613	0.287133
TRAJ11	0.388909	-0.26475	0.021458	-0.23221	0.584411	-0.30542	0.823374	-0.26233	0.322679	-0.67241	-0.05065	-0.11152	-0.34192	-0.24725	-0.24332	-0.38986	0.205681	0.494749
ADAM8	0.39175	-0.37313	0.004679	-0.10909	0.364236	-0.36789	0.749918	-0.18505	0.313124	-0.38292	0.14243	-0.09301	-0.35735	-0.19134	-0.29846	-0.48476	0.557429	0.304408
TRBV29-1	0.394428	-0.27208	0.125192	-0.2095	0.491342	-0.20978	0.769362	-0.20162	0.427963	-0.60225	-0.02467	-0.16917	-0.25701	-0.34983	-0.27216	-0.3795	0.323014	0.632965
SEMA4D	0.396427	-0.25609	-0.13874	0.00842	0.638363	-0.5315	0.771895	-0.08447	-0.03102	-0.47164	-0.04692	-0.16939	-0.23005	-0.12671	-0.1993	-0.40564	0.199473	0.348948
APOBEC3F	0.399399	-0.34232	0.068646	0.360732	0.285522	-0.43374	0.105062	0.373678	-0.66227	-0.07849	-0.00685	-0.3716	0.226142	0.229088	-0.37797	0.326761	-0.31044	0.097236
CXCL14	0.40109	-0.22173	0.017583	0.53236	0.242604	-0.74741	0.380111	0.467473	-0.37851	-0.2635	0.246571	-0.37626	0.238977	0.194829	-0.30452	-0.01882	-0.01202	0.118246
GIMAP2	0.401939	-0.25128	0.038851	0.169505	0.307382	-0.72557	0.538173	0.0707	-0.15151	-0.4341	0.308381	-0.19264	0.027857	0.103359	-0.28576	-0.29563	0.073864	0.306763
AP4B1-AS1	0.405918	-0.23706	0.167559	-0.01815	0.241053	-0.70992	0.705304	-0.11205	0.096342	-0.53496	0.514598	-0.09743	-0.03768	0.084895	-0.38497	-0.29971	0.092897	0.11304
TRAF5	0.406152	-0.21565	0.175712	0.087917	0.345761	-0.76291	0.573872	0.020544	-0.14573	-0.47173	0.272354	-0.06454	-0.12233	0.082746	-0.22336	-0.17291	0.009056	0.19027
CCR2	0.406173	-0.34166	0.196144	0.03932	0.110929	-0.72869	0.469004	-0.05304	-0.05765	-0.48902	0.539459	-0.15931	0.01848	0.247718	-0.46917	-0.13696	0.089138	0.104282
CCL5	0.406666	-0.17152	0.192465	0.424616	-0.20511	-0.77855	0.282797	0.319741	0.024544	-0.36026	0.600906	-0.18134	0.217863	0.250023	-0.32011	-0.02416	0.205523	-0.06365
TMEM66	0.410467	-0.45957	-0.02498	0.075683	0.395971	-0.40057	0.765347	0.004199	0.358464	-0.51858	0.134009	-0.32879	-0.32888	-0.0496	-0.4549	-0.35767	0.524051	0.288908
ITGA4	0.412883	-0.31922	0.075129	0.030003	0.303017	-0.77684	0.614892	-0.09192	0.002001	-0.58965	0.400927	-0.22716	-0.21276	0.286175	-0.32839	-0.32747	0.119347	0.111661
BTN3A3	0.413059	-0.33993	0.116761	0.283661	0.252168	-0.75104	0.430818	0.208497	-0.09373	-0.49892	0.230231	-0.12182	-0.12428	0.172059	-0.32483	-0.14358	0.03681	0.328558
SUSD3	0.414397	-0.2737	0.017172	0.010305	0.423233	-0.64498	0.665438	-0.08913	-0.12747	-0.42133	0.238257	-0.24384	-0.13912	0.074694	-0.25076	-0.31235	0.192485	0.167894
IGHGP	0.41541	-0.27283	0.273854	0.495801	-0.21494	-0.71408	0.058722	0.409443	-0.39278	-0.05351	0.521271	-0.14585	0.182518	0.41802	-0.37864	0.386536	-0.15984	-0.30662
WIPF1	0.415901	-0.28051	0.138467	0.144864	0.308278	-0.79139	0.63402	0.054201	-0.05607	-0.49151	0.411395	-0.12567	-0.02812	0.116153	-0.38394	-0.25539	0.032199	0.22124
TPP1	0.416002	-0.39123	0.09632	0.31943	0.077406	-0.82663	0.392061	0.194168	-0.21782	-0.3231	0.508862	-0.09074	-0.00545	0.332324	-0.45265	-0.13193	-0.00311	0.04151
BTN2A2	0.41667	-0.16509	0.20399	0.045616	0.299806	-0.78743	0.618298	-0.02771	-0.05566	-0.54096	0.371909	-0.15067	-0.06759	0.1189	-0.23496	-0.17455	0.069301	0.082196
CD8B	0.41769	-0.30807	0.122476	0.177341	0.248467	-0.63988	0.526283	0.108352	-0.02879	-0.38438	0.225477	-0.05609	-0.04558	-0.06378	-0.35212	-0.10873	0.265486	0.263368
ANKRD44-IT1	0.420303	-0.36875	0.138828	0.049533	0.505653	-0.64633	0.617031	-0.02664	-0.18183	-0.41396	0.146085	-0.13907	-0.22986	0.074485	-0.37368	-0.07522	-0.06433	0.276008
LOC100128670	0.423062	-0.15695	-0.18654	0.133474	0.455229	-0.5325	0.620716	0.028252	0.08656	-0.46103	-0.05158	-0.23626	-0.10932	-0.12218	-0.07912	-0.47354	0.35179	0.354798
CERKL	0.424036	-0.3272	0.119686	-0.03557	0.284287	-0.74145	0.630972	-0.15431	0.016416	-0.59835	0.478715	-0.3351	-0.18124	0.341	-0.37153	-0.30508	0.098259	0.064988
CD226	0.424163	-0.2224	0.050579	0.000157	0.491656	-0.61539	0.771564	-0.07593	0.05457	-0.54515	0.142703	-0.05453	-0.2107	-0.10405	-0.21373	-0.35822	0.096248	0.340772
TRIM22	0.426502	-0.19112	0.319939	0.520302	0.045672	-0.70231	0.20201	0.522701	-0.40102	-0.24185	0.287781	-0.08484	0.306741	0.090109	-0.32875	0.335786	-0.27388	0.188275

PTPRC	0.426801	-0.33405	0.024321	0.043199	0.366556	-0.72717	0.675774	-0.06533	0.001445	-0.52746	0.341151	-0.19792	-0.16248	0.148112	-0.34577	-0.4196	0.177465	0.218676
IGLV2-23	0.431927	-0.27646	0.065806	0.298823	-0.0507	-0.4243	0.540062	0.22994	0.291869	-0.41451	0.37147	-0.15101	0.049719	-0.07648	-0.37331	-0.14017	0.422497	0.116216
C16orf54	0.432972	-0.24327	-0.02468	0.130796	0.531073	-0.62024	0.741891	0.059736	0.16899	-0.59304	0.028991	-0.23475	-0.21105	-0.09046	-0.21853	-0.42547	0.263131	0.429028
EVI2A	0.434776	-0.29054	0.138366	0.014249	0.302491	-0.65076	0.728234	-0.06965	0.075909	-0.44536	0.407669	-0.1815	-0.12669	0.024533	-0.32659	-0.383	0.244793	0.206923
STAT1	0.43553	-0.14397	0.335371	0.242402	-0.22789	-0.67822	0.207487	0.199329	-0.26257	-0.28701	0.662763	-0.22216	0.394767	0.255392	-0.31474	0.141867	-0.01197	-0.08997
BCL11B	0.435767	-0.28457	-0.00474	0.105728	0.480167	-0.68256	0.694443	0.012247	0.034888	-0.54392	0.121487	-0.2202	-0.21825	0.018609	-0.24599	-0.36571	0.232211	0.319707
RNA5SP321	0.436901	-0.09289	0.118152	0.289971	0.288393	-0.84004	0.403989	0.207661	-0.10084	-0.49181	0.132277	-0.08893	-0.08483	0.177946	-0.11182	-0.04857	-0.10103	0.040532
EVI2B	0.437241	-0.35878	0.135014	0.117448	0.315583	-0.64912	0.683401	0.045587	0.111844	-0.46207	0.284496	-0.09348	-0.19037	-0.04648	-0.36768	-0.30581	0.296	0.326291
TRAC	0.437819	-0.37746	-0.02825	0.200885	0.447623	-0.63746	0.644097	0.105852	-0.03644	-0.52659	0.152556	-0.3644	-0.1186	0.101135	-0.40162	-0.21436	0.198248	0.302497
ADAMDEC1	0.437981	-0.41934	0.045399	0.017055	0.484285	-0.59961	0.690438	-0.08191	0.053489	-0.45532	0.165333	-0.14749	-0.28536	0.023802	-0.41203	-0.32021	0.136852	0.360597
AMICA1	0.438978	-0.32099	0.159287	0.119834	0.338137	-0.85369	0.525906	0.028408	-0.08418	-0.53227	0.294149	-0.01675	-0.21192	0.223676	-0.34768	-0.18626	-0.08544	0.165957
KIAA1211	0.444723	-0.49551	0.29149	0.37371	0.028179	-0.64095	0.218672	0.306447	-0.31817	-0.15518	0.34855	-0.23676	-0.21927	0.393667	-0.39474	0.211896	-0.01614	0.011748
IGHV4OR15-8	0.445747	-0.21991	0.123192	-0.16933	0.388884	-0.31713	0.892055	-0.21496	0.489327	-0.54602	0.194879	-0.19367	-0.29008	-0.24016	-0.23441	-0.4641	0.359556	0.311192
FYB	0.446854	-0.32054	0.063082	0.085906	0.383042	-0.78193	0.612807	-0.0088	-0.09437	-0.54523	0.323457	-0.19194	-0.09017	0.184494	-0.36013	-0.34139	0.013736	0.261698
SLC15A3	0.448191	-0.25244	0.089304	0.245169	0.21802	-0.80404	0.478847	0.136529	-0.39024	-0.25337	0.383203	-0.09841	-0.05965	0.299447	-0.25343	-0.05921	-0.13625	-0.19372
LINC00426	0.448334	-0.38897	-0.11283	0.111549	0.542667	-0.66689	0.704236	-0.02505	-0.03764	-0.46861	0.112238	-0.25452	-0.29411	0.142374	-0.34491	-0.3699	0.121222	0.217357
ANXA2R	0.448347	-0.2626	0.32712	0.305258	0.249622	-0.80127	0.453937	0.258241	-0.0955	-0.43739	0.327352	-0.17146	0.036923	0.138789	-0.42851	0.132671	-0.12924	0.1933
LCK	0.448557	-0.28913	0.019455	-0.09842	0.470053	-0.47717	0.704054	-0.14966	0.143616	-0.55561	0.039369	-0.17156	-0.22755	-0.15963	-0.22296	-0.40968	0.36378	0.430773
COMP	0.449363	-0.3114	0.248651	-0.061	0.160192	-0.59289	0.56303	-0.11262	0.175323	-0.3806	0.359511	0.162186	-0.11002	-0.13483	-0.38793	-0.26055	0.234469	0.258074
LPAR5	0.453085	-0.38128	0.130485	0.210873	0.304587	-0.76021	0.440782	0.123121	-0.28992	-0.45556	0.375844	-0.45378	-0.07812	0.524594	-0.45665	0.02043	-0.24114	-0.08489
CD48	0.45594	-0.27566	0.107849	-0.06126	0.415006	-0.70126	0.695642	-0.13912	0.099316	-0.60634	0.314572	-0.21058	-0.10354	0.067997	-0.34411	-0.43687	0.107901	0.339829
STARD5	0.456085	-0.53941	0.026329	0.474669	0.144249	-0.56056	0.433134	0.381493	-0.40429	-0.06553	0.324272	-0.39045	-0.14568	0.376892	-0.48968	0.163916	0.106564	-0.28301
DAPP1	0.457865	-0.39545	-0.01122	0.065794	0.378365	-0.72503	0.615321	-0.07212	-0.15319	-0.39359	0.324558	-0.24646	-0.22883	0.275213	-0.34904	-0.35791	0.075474	0.095456
TRAF3IP3	0.457962	-0.33705	0.073987	-0.0996	0.596165	-0.56261	0.672616	-0.16863	-0.14939	-0.46182	0.083713	-0.21438	-0.19741	0.020297	-0.32544	-0.24237	-0.06869	0.375873
APOBEC3G	0.458132	-0.21233	0.25741	0.165733	0.163741	-0.71991	0.37671	0.106813	-0.22669	-0.3993	0.330198	-0.19395	0.089197	0.141295	-0.28034	0.069178	-0.01462	0.148402
CD3G	0.458376	-0.20691	0.037211	0.073737	0.437505	-0.68851	0.675907	-0.01586	0.109144	-0.60646	0.150688	-0.26866	-0.15007	0.013611	-0.19623	-0.38083	0.221835	0.344538
IKZF1	0.458691	-0.37931	-0.03166	-0.03179	0.506291	-0.61564	0.725539	-0.13037	-0.06126	-0.47661	0.179724	-0.20119	-0.23528	0.059482	-0.33263	-0.42491	0.152551	0.285715
EEF1B2P1	0.462788	-0.06902	0.361376	0.377922	0.092518	-0.74283	0.279302	0.363478	-0.27652	-0.36781	0.362871	-0.1724	0.40145	0.10085	-0.31339	0.276537	-0.38314	0.183313
TLR5	0.464957	-0.28869	0.166825	0.343034	0.277773	-0.88032	0.320663	0.259314	-0.29332	-0.42951	0.265429	-0.19801	0.001536	0.361377	-0.35842	0.022219	-0.30793	0.14416
CD96	0.465307	-0.29459	0.008153	0.027307	0.504199	-0.69679	0.759205	-0.0632	0.085634	-0.61835	0.184399	-0.25294	-0.19876	0.047256	-0.30349	-0.42209	0.19234	0.299989
GPR174	0.468628	-0.13652	-0.01009	0.329398	0.353442	-0.70388	0.573627	0.248522	0.038917	-0.59684	0.08645	-0.29593	0.007063	0.016015	-0.16009	-0.21179	0.142392	0.296319
ST8SIA4	0.469229	-0.35149	0.131792	0.061541	0.170359	-0.65649	0.63425	-0.02477	0.095731	-0.42145	0.457158	-0.15667	-0.10942	0.069602	-0.35925	-0.41473	0.324563	0.203429
SORL1	0.46943	-0.36699	0.202096	0.082915	0.392648	-0.67992	0.600435	0.032746	0.060514	-0.53484	0.17141	-0.09875	-0.20981	-0.01332	-0.37869	-0.18298	0.138611	0.41356
TRAT1	0.47224	-0.29955	-0.06575	0.047986	0.592788	-0.49611	0.82272	-0.01845	0.191136	-0.52185	-0.02706	-0.27112	-0.26782	-0.16246	-0.24393	-0.47305	0.332425	0.40984
LINC00861	0.472498	-0.29689	-0.01374	-0.05528	0.602629	-0.49975	0.675267	-0.13488	-0.19417	-0.40725	0.015227	-0.27163	-0.15824	-0.02515	-0.24049	-0.27308	-0.02448	0.377924
PTPN22	0.473386	-0.36255	0.101946	0.081329	0.266601	-0.65599	0.728554	-0.01223	0.187511	-0.48551	0.339251	-0.07631	-0.25332	-0.00836	-0.34618	-0.38819	0.351939	0.214933
EPSTI1	0.475462	-0.21724	0.210709	0.357768	0.027754	-0.77278	0.446333	0.280431	-0.19904	-0.38277	0.529682	-0.38302	0.190078	0.255493	-0.31742	0.009688	0.119646	-0.03222
RGS1	0.476334	-0.34229	0.022967	0.26604	0.191746	-0.70452	0.630644	0.133379	0.032751	-0.35426	0.35108	-0.26111	-0.2424	0.171326	-0.25957	-0.30622	0.396489	-0.01219
LY75	0.476901	-0.25619	0.250964	0.000924	0.399387	-0.68	0.589936	-0.0301	-0.0387	-0.5197	0.223361	-0.15536	-0.03535	-0.03676	-0.31831	-0.18895	0.033404	0.424327
CYTIP	0.477594	-0.38407	0.000266	0.111774	0.341544	-0.63776	0.703947	0.016731	0.104062	-0.45808	0.251793	-0.21257	-0.23789	0.039744	-0.32463	-0.45627	0.383581	0.235423
SAMD3	0.481111	-0.39856	0.018445	0.023119	0.538083	-0.59354	0.773045	-0.06336	0.003533	-0.53381	0.152235	-0.33492	-0.23824	0.050465	-0.39172	-0.28866	0.192578	0.281531
MNDA	0.481254	-0.34538	0.210915	0.032987	0.270917	-0.70975	0.621145	-0.03199	0.081212	-0.58606	0.38655	-0.24128	-0.10753	0.108708	-0.39198	-0.27694	0.206613	0.308257
FAP	0.481261	-0.29232	0.351081	0.13535	0.007573	-0.66002	0.391285	0.078657	-0.07529	-0.25307	0.399364	0.107868	-0.07848	-0.00314	-0.28896	0.011997	0.136231	0.137401
KCNA3	0.483811	-0.1932	0.006124	0.03137	0.496147	-0.6753	0.718452	-0.06392	-0.06786	-0.4544	0.088826	-0.12497	-0.20385	-0.04076	-0.14186	-0.30163	0.154652	0.138975
CD3E	0.483831	-0.48565	0.094412	0.158685	0.547725	-0.64342	0.691882	0.104746	-0.08388	-0.4583	0.116703	-0.17349	-0.18009	0.016674	-0.56023	-0.10498	0.003285	0.339596
HLA-DMB	0.485201	-0.46763	0.118444	0.102917	0.245855	-0.7547	0.6322	-0.01517	-0.05012	-0.43466	0.453094	-0.22128	-0.27307	0.316527	-0.47245	-0.1967	0.177176	-0.07901

LEF1	0.490744	-0.36265	-0.05856	-0.05494	0.591401	-0.49933	0.770884	-0.13371	0.005712	-0.44673	0.021798	-0.17436	-0.2303	-0.12699	-0.30699	-0.43778	0.18803	0.416733
UBE2L6	0.492165	-0.14925	0.122529	0.390137	0.025399	-0.84509	0.312801	0.306943	-0.34343	-0.27906	0.451027	-0.17132	0.305561	0.216755	-0.23709	-0.09775	-0.04976	0.015268
THEMIS	0.492534	-0.21446	0.141208	0.092318	0.397163	-0.66284	0.686267	0.051409	0.15682	-0.62836	0.148183	-0.17369	-0.12171	-0.10311	-0.21228	-0.35082	0.214775	0.451035
TRAJ15	0.493076	-0.31502	0.067949	-0.24257	0.510873	-0.50094	0.849546	-0.30794	0.180152	-0.61973	0.221454	-0.28152	-0.23421	-0.03201	-0.33296	-0.45352	0.228311	0.275573
LY9	0.495642	-0.21675	-0.03623	0.096365	0.590051	-0.64899	0.736779	0.010418	-0.00095	-0.53428	0.007574	-0.29729	-0.16805	-0.03823	-0.18166	-0.36428	0.134975	0.333802
STX7	0.499106	-0.36466	0.289789	0.243271	0.279267	-0.85154	0.431682	0.19206	-0.1778	-0.42767	0.278954	0.011879	-0.13451	0.203152	-0.43046	0.049767	-0.20963	0.146499
C5orf58	0.500763	-0.36582	0.136517	0.104332	0.332514	-0.67432	0.734091	0.025755	0.007401	-0.44769	0.345435	-0.25021	-0.13528	0.036286	-0.39335	-0.24607	0.288298	0.161629
RPL23AP7	0.502422	-0.48574	0.342554	0.221492	-0.00653	-0.55621	0.20674	0.200427	-0.4672	-0.11663	0.396655	-0.05277	0.002179	0.289553	-0.51733	0.365443	-0.24527	-0.09521
IKZF3	0.503211	-0.20381	-0.00825	0.181969	0.436724	-0.78175	0.687624	0.066141	-0.06591	-0.50236	0.157607	-0.15824	-0.16796	0.061164	-0.16441	-0.31729	0.092149	0.155504
HERPUD1	0.505474	-0.13501	0.280471	0.448909	0.011861	-0.74707	0.402939	0.36161	-0.08266	-0.2306	0.359886	-0.16212	0.041122	0.069893	-0.15583	0.088904	0.111488	0.01049
LOC101060038	0.505971	-0.2228	0.055401	0.008457	0.334947	-0.58896	0.710804	-0.07359	0.284821	-0.53937	0.168045	-0.08993	-0.16747	-0.15177	-0.22668	-0.42611	0.357546	0.301013
TRBC2	0.506202	-0.38772	0.041099	0.129129	0.455974	-0.6456	0.645783	0.050713	0.099911	-0.61058	0.1075	-0.33843	-0.24544	0.102946	-0.35493	-0.29568	0.191279	0.369767
TAP2	0.508282	-0.26943	0.360509	0.355925	-0.06926	-0.76555	0.233491	0.317103	-0.36922	-0.28482	0.553704	-0.17355	0.28065	0.287302	-0.39932	0.176819	-0.25862	0.080287
OLFML2B	0.508415	-0.39321	0.383751	0.437917	-0.19089	-0.75177	0.203752	0.393543	-0.15024	-0.26656	0.618023	-0.30997	0.205952	0.320315	-0.50964	0.136745	0.11482	0.077796
PLB1	0.508429	-0.33338	0.216907	0.215298	0.22315	-0.77896	0.601874	0.115074	-0.05272	-0.45722	0.448635	-0.32387	-0.06424	0.218282	-0.40768	-0.08165	0.108793	0.083218
IGHV3-30	0.508522	-0.2684	0.22729	0.230901	0.130352	-0.71498	0.672644	0.145741	0.034669	-0.30872	0.443904	-0.09963	-0.0918	0.004574	-0.3276	-0.08104	0.308336	-0.09968
CARD16	0.509285	-0.37079	0.05007	0.120709	0.245393	-0.70539	0.670911	0.008905	0.081555	-0.52854	0.414721	-0.30605	-0.0844	0.158364	-0.41882	-0.3484	0.291279	0.147888
CXCR4	0.512191	-0.41325	0.042974	0.220726	0.303143	-0.66714	0.636683	0.126742	0.12285	-0.40243	0.193382	-0.14602	-0.26694	0.018005	-0.35667	-0.31931	0.385942	0.203358
CNOT6L	0.515498	-0.55802	0.094603	0.131055	0.381833	-0.48016	0.669743	0.102836	0.137195	-0.41374	0.158595	-0.16801	-0.19322	-0.09742	-0.56598	-0.31584	0.284212	0.487263
IL2RG	0.517442	-0.36445	-0.01541	0.15152	0.424969	-0.6198	0.750533	0.061525	-0.0126	-0.44063	0.220183	-0.28419	-0.07813	-0.02308	-0.37851	-0.36178	0.249916	0.293738
BLNK	0.521688	-0.42314	0.134484	0.053204	0.311359	-0.71755	0.461054	-0.04284	-0.29158	-0.37118	0.313019	-0.26534	-0.24774	0.426705	-0.367	-0.09665	-0.15967	-0.07217
CD2	0.522614	-0.27095	0.059946	0.169683	0.34151	-0.71813	0.667501	0.071514	0.096027	-0.53929	0.18978	-0.22189	-0.2186	0.049002	-0.21752	-0.30666	0.282215	0.19383
GGTA1P	0.524107	-0.32388	0.34758	0.279804	0.218076	-0.74735	0.53265	0.251313	-0.09296	-0.49943	0.463659	-0.56036	0.027452	0.359067	-0.46576	0.089479	-0.07244	0.001716
CD74	0.524549	-0.34258	0.223436	0.344799	0.0349	-0.81185	0.441482	0.259006	-0.14207	-0.36404	0.469039	-0.08708	0.026166	0.180138	-0.40061	-0.01267	0.093239	0.047968
ARHGAP15	0.527469	-0.54044	0.167341	0.186035	0.426603	-0.68235	0.636675	0.139965	-0.12415	-0.42701	0.195117	-0.17209	-0.22931	0.080491	-0.5426	-0.08561	0.102236	0.27932
HLA-DOA	0.528145	-0.32254	0.228746	0.258412	0.006478	-0.71652	0.544535	0.151543	-0.0077	-0.29593	0.501321	-0.15535	-0.10933	0.154681	-0.33802	-0.03021	0.293789	-0.14845
SLAMF6	0.528688	-0.30273	-0.01001	0.103634	0.52547	-0.7126	0.748194	0.006061	-0.05029	-0.48557	0.122315	-0.15703	-0.17996	-0.0108	-0.28151	-0.36711	0.096799	0.2737
IGLC7	0.531251	-0.31226	0.2713	0.548781	-0.021	-0.54677	0.134862	0.56559	-0.50363	-0.02597	0.218966	-0.24021	0.317715	0.105378	-0.39184	0.457647	-0.0877	-0.04302
RHOH	0.533919	-0.26388	0.02523	0.015404	0.416053	-0.54544	0.815409	-0.06627	0.137679	-0.52294	0.179674	-0.30529	-0.18754	-0.09221	-0.20683	-0.42263	0.368972	0.267057
SULF1	0.536149	-0.38337	0.305346	0.229478	-0.17886	-0.52899	0.41614	0.161609	-0.0214	-0.25852	0.568099	-0.28196	-0.01013	0.1848	-0.40139	0.124489	0.359026	-0.1584
SH2D1A	0.536571	-0.35626	0.005802	0.180019	0.463376	-0.59554	0.785505	0.105473	0.053679	-0.52343	0.175871	-0.42008	-0.10037	-0.00406	-0.3746	-0.31461	0.26254	0.301524
ZNF683	0.542602	-0.46291	0.387486	0.235854	-0.08702	-0.67736	0.274934	0.212333	-0.05038	-0.40422	0.553774	-0.24874	0.143153	0.268209	-0.62216	0.120344	0.060502	0.143441
SELL	0.542953	-0.26928	0.147942	-0.01273	0.528225	-0.5907	0.760244	-0.03528	0.122829	-0.62203	0.112509	-0.19662	-0.03924	-0.16644	-0.36568	-0.30365	0.053477	0.504702
DMP1	0.547771	-0.01824	0.296779	0.186392	-0.07532	-0.57093	0.459181	0.123959	-0.0776	-0.34014	0.467034	-0.28396	0.218621	0.02342	-0.07002	0.053676	0.122375	-0.04902
IKZF2	0.550856	-0.41448	0.085974	0.035593	0.48173	-0.52802	0.735958	-0.02568	0.078953	-0.45588	0.098241	-0.29869	-0.2455	-0.07425	-0.35781	-0.31136	0.290884	0.392012
STAMBPL1	0.552764	-0.37415	0.21253	0.101541	0.25913	-0.70055	0.468521	0.04746	-0.338	-0.33771	0.366878	-0.26632	0.028065	0.209561	-0.37501	-0.1284	-0.08549	0.205871
ANKRD22	0.553279	-0.25007	0.27005	0.037185	0.231799	-0.68204	0.43773	0.022994	-0.12607	-0.54766	0.331676	-0.29405	0.20124	0.089721	-0.36541	-0.12886	-0.03944	0.371518
TRGV10	0.559537	-0.37503	0.236687	0.032434	0.235513	-0.59695	0.626714	0.013904	0.136416	-0.54964	0.288664	-0.06758	-0.02767	-0.1267	-0.45338	-0.20471	0.250909	0.383799
IFNG-AS1	0.563965	-0.25037	0.089115	0.064262	0.27521	-0.65315	0.690548	-0.04902	-0.05812	-0.41215	0.388293	-0.32696	-0.05987	0.185694	-0.31726	-0.15737	0.053333	-0.15595
TMEM156	0.564736	-0.29008	0.027498	0.013944	0.383012	-0.54667	0.771116	-0.05781	0.138704	-0.50055	0.140452	-0.16158	-0.24085	-0.12374	-0.18021	-0.47282	0.341179	0.305087
CD3D	0.568541	-0.18264	0.07107	0.088236	0.465465	-0.58881	0.750932	0.053144	0.079324	-0.56213	0.048134	-0.21527	-0.03846	-0.22244	-0.1743	-0.33549	0.235288	0.415339
GBP5	0.571342	-0.2484	0.14776	0.106098	0.06396	-0.66978	0.605067	0.008106	0.062313	-0.45223	0.520967	-0.33112	0.091472	0.111021	-0.31918	-0.27191	0.319352	0.035516
LOC100996286	0.571817	-0.2993	0.121466	0.038024	0.425852	-0.61429	0.579788	-0.03013	-0.15969	-0.34995	0.183166	-0.26655	0.018828	0.004222	-0.32626	-0.2449	-0.03423	0.353741
APOBEC3D	0.572041	-0.3713	0.106012	0.16444	0.353541	-0.63615	0.591773	0.099123	-0.39108	-0.23124	0.321755	-0.26501	0.123783	0.120846	-0.47489	-0.03463	-0.15694	0.03466
C11orf80	0.572625	-0.44953	0.339402	0.513156	0.105321	-0.77525	0.335332	0.456366	-0.33575	-0.14924	0.353129	-0.2792	-0.05875	0.304611	-0.46248	0.287128	-0.08336	-0.04082
RAB39B	0.572693	-0.39044	0.280175	-0.08352	0.453106	-0.51621	0.764204	-0.09746	0.027503	-0.44373	0.236036	-0.19221	-0.11329	-0.14116	-0.44862	-0.2	0.068112	0.41574

ICOS	0.573105	-0.3029	0.163861	0.10987	0.281315	-0.61672	0.694507	0.053462	-0.06681	-0.41411	0.291155	-0.23774	-0.05182	-0.05355	-0.28432	-0.19894	0.266257	0.194105
CD8A	0.574275	-0.26175	0.18243	0.285703	0.131291	-0.65487	0.612528	0.223173	0.135232	-0.44618	0.305737	-0.19691	-0.01285	-0.08939	-0.26511	-0.20797	0.369815	0.263897
BTBD19	0.574435	-0.37883	0.086035	0.234284	0.255579	-0.47603	0.607119	0.190208	-0.28104	-0.14799	0.161785	-0.13793	-0.12242	-0.00257	-0.33482	0.101226	0.118216	-0.20697
FAM26F	0.576946	-0.49825	0.219959	0.276909	-0.04153	-0.64532	0.445932	0.203087	-0.06713	-0.29965	0.563851	-0.39235	0.039513	0.250623	-0.5248	-0.11425	0.33811	0.069185
NELL2	0.577558	-0.40302	0.183946	-0.12414	0.354759	-0.47647	0.71872	-0.15872	0.217037	-0.62512	0.26385	-0.49358	-0.19821	0.060708	-0.38316	-0.34621	0.345572	0.328149
IGKV1-5	0.578202	-0.2293	0.119575	0.315511	0.11575	-0.77493	0.577607	0.201381	-0.11964	-0.33089	0.41754	-0.1999	0.0051	0.173718	-0.27807	-0.05654	0.104886	-0.20743
CD1C	0.578654	-0.31921	0.414366	0.230556	0.146074	-0.6708	0.260436	0.249238	-0.37538	-0.30782	0.265325	-0.0721	0.032201	0.235544	-0.37259	0.283889	-0.46924	0.058032
SFRP4	0.579174	-0.1287	0.141953	0.308607	0.216564	-0.67754	0.698604	0.227475	-0.01475	-0.3431	0.235268	-0.16047	-0.09763	-0.07019	-0.08744	-0.09812	0.233154	-0.07244
GPR141	0.584728	-0.36241	0.48715	0.096643	-0.00829	-0.67017	0.41204	0.063932	0.053464	-0.36627	0.48441	-0.09668	-0.07328	0.088335	-0.39763	0.012363	0.106202	0.214053
CD69	0.589039	-0.29189	0.045178	0.246175	0.290734	-0.66269	0.629681	0.157967	-0.03131	-0.37646	0.214941	-0.30399	-0.01449	-0.02184	-0.26374	-0.28843	0.307599	0.233048
TAPSAR1	0.592365	-0.25227	0.22103	0.210829	0.302256	-0.82233	0.541082	0.154146	-0.14035	-0.53615	0.31522	-0.35043	0.048776	0.181712	-0.3002	-0.15306	-0.03959	0.240269
IGKV3-11	0.593479	-0.31924	0.235681	0.343949	0.143127	-0.83349	0.615695	0.262364	-0.05157	-0.38626	0.439818	-0.06038	-0.0471	0.109413	-0.39597	-0.06072	0.035417	-0.01965
FCRL3	0.595777	-0.1515	0.124804	0.124047	0.389109	-0.71082	0.461397	0.078573	-0.30553	-0.45366	0.113708	-0.28823	0.107456	0.088647	-0.13672	-0.16336	-0.18251	0.288038
TMEM154	0.60082	-0.20948	0.249316	0.043739	0.412705	-0.64149	0.591173	0.014211	-0.10534	-0.40641	0.129411	-0.05395	0.027589	-0.16551	-0.24664	-0.16958	-0.12936	0.461323
IL2RB	0.602459	-0.35223	0.221974	0.234437	0.087654	-0.71477	0.565311	0.151132	-0.03748	-0.38067	0.460812	-0.29428	0.000107	0.113396	-0.35998	-0.15546	0.282772	0.120585
GAPT	0.604036	-0.40789	0.424908	0.200126	0.060658	-0.67002	0.451346	0.160591	-0.19763	-0.35004	0.594099	-0.52554	0.098983	0.333576	-0.49591	0.080294	-0.0606	0.107971
GPR114	0.607176	-0.3061	0.072179	0.257	0.373948	-0.73011	0.591782	0.177781	-0.01761	-0.45079	0.096697	-0.24798	-0.17741	0.070633	-0.2449	-0.218	0.150626	0.174662
IGKV3-15	0.611643	-0.24995	0.24156	0.421461	0.007738	-0.74456	0.444189	0.360322	-0.28222	-0.13902	0.414411	-0.04929	0.125064	0.101693	-0.32772	0.160937	-0.02662	-0.26185
ZBP1	0.613595	-0.40386	0.417285	0.212632	0.069259	-0.74664	0.418746	0.191535	-0.28463	-0.26713	0.479001	0.000412	0.081233	0.126193	-0.51146	0.156538	-0.17806	0.062145
SLAMF7	0.621125	-0.26955	0.262144	0.142233	0.084815	-0.81557	0.538467	0.073354	-0.11144	-0.45141	0.538061	-0.20728	0.074336	0.22325	-0.35129	-0.1479	0.02322	-0.0518
IGHA2	0.622688	-0.25571	0.486791	0.124798	-0.09242	-0.69085	0.440775	0.07723	0.031532	-0.37656	0.578457	-0.23693	-0.01633	0.192083	-0.30427	0.080276	0.107017	-0.07381
IGLV3-19	0.629493	-0.31329	0.396788	0.288668	0.056399	-0.72061	0.562701	0.249788	-0.03836	-0.23938	0.471011	-0.14432	0.065121	-0.03523	-0.39912	0.02513	0.20146	0.068634
IGLV3-21	0.63029	-0.39156	0.42002	0.45651	-0.08512	-0.62252	0.347914	0.454089	-0.12742	-0.25468	0.440924	-0.27641	0.235659	0.032773	-0.5068	0.259835	0.138434	0.191729
ANKRD36BP2	0.630775	-0.39997	0.276522	0.19684	0.364878	-0.68825	0.63186	0.146019	-0.33453	-0.27955	0.326138	-0.30899	-0.05	0.16144	-0.43766	0.06049	-0.19559	0.062901
TRIM59	0.632109	-0.51469	0.340288	0.086015	0.181084	-0.52574	0.617857	0.051668	0.04276	-0.25436	0.296611	-0.06925	-0.29807	-0.07543	-0.41424	-0.11703	0.287426	0.251642
IGHV3-49	0.632126	-0.32712	0.257523	0.32351	0.238771	-0.75973	0.639657	0.242682	-0.20635	-0.2813	0.410084	-0.3645	-0.00645	0.163239	-0.37796	0.029129	0.019487	-0.04475
IGLV6-57	0.64249	-0.29431	0.174305	0.103639	0.248025	-0.62439	0.724526	0.035134	-0.11591	-0.36769	0.382842	-0.29839	-0.0356	0.098119	-0.34367	-0.07033	0.080937	-0.16987
IGKC	0.649685	-0.32949	0.320674	0.129741	0.116633	-0.72329	0.534456	0.052738	-0.17876	-0.33329	0.52256	-0.32166	0.060136	0.218377	-0.40274	-0.01301	-0.02881	-0.01006
IGHV5-51	0.651228	-0.36341	0.271062	0.365086	-0.01656	-0.59898	0.536497	0.334173	-0.00919	-0.32621	0.435543	-0.2472	0.181165	-0.03556	-0.47552	0.064485	0.274566	0.078909
IGHG2	0.652595	-0.41484	0.41941	0.474536	-0.24908	-0.56977	0.18063	0.434442	-0.23492	0.017693	0.446502	-0.22509	0.044548	0.189452	-0.36784	0.33246	0.148786	-0.1305
IGHG3	0.654023	-0.26327	0.377923	0.528495	-0.02879	-0.67037	0.374431	0.504562	-0.31753	-0.05399	0.326634	-0.03249	0.120757	-2.57E-06	-0.30113	0.358102	-0.09501	-0.12532
PARP15	0.655085	-0.33421	0.274029	0.175352	0.438353	-0.71818	0.570393	0.163231	-0.32035	-0.33328	0.124847	-0.08855	-0.03772	-0.02495	-0.32873	-0.0248	-0.23101	0.299996
IGHV3-33	0.659673	-0.35983	0.356051	0.298591	0.016492	-0.71607	0.474048	0.246769	-0.16757	-0.26482	0.519486	-0.30779	0.170084	0.120497	-0.45965	0.080271	0.111805	0.051
IGLV3-25	0.666738	-0.39865	0.466621	0.42781	-0.17769	-0.59536	0.364245	0.452393	-0.0865	-0.25908	0.527892	-0.27402	0.263744	0.051776	-0.53985	0.274637	0.177916	0.033384
IGLV2-8	0.673903	-0.30644	0.275605	-0.23596	0.170532	-0.38757	0.635005	-0.23813	0.197936	-0.50426	0.336867	-0.15297	0.073462	-0.17778	-0.38859	-0.30001	0.168089	0.346261
MIAT	0.675997	-0.23874	0.155419	0.023617	0.315382	-0.63161	0.751765	-0.04945	-0.07276	-0.38459	0.296392	-0.15983	-0.01529	-0.07308	-0.26158	-0.22075	0.077855	0.05863
TP53INP1	0.681258	-0.50993	0.220988	0.224265	0.395195	-0.51046	0.673456	0.209133	-0.14437	-0.25963	0.062943	-0.20662	-0.21148	-0.14273	-0.40961	-0.01349	0.192743	0.310029
IGKJ3	0.688997	-0.30332	0.211756	0.265112	0.285335	-0.7372	0.537502	0.190949	-0.32267	-0.24672	0.319439	-0.31016	0.098197	0.156317	-0.35157	-0.02978	-0.21532	0.07444
RAB30	0.700488	-0.31894	0.476267	0.224734	-0.01063	-0.52441	0.376427	0.273205	-0.27675	-0.27925	0.379197	-0.30573	0.218287	0.0164	-0.33988	0.207407	-0.0161	0.175205
IGLV3-1	0.702306	-0.38775	0.361048	0.423654	0.070684	-0.74217	0.588989	0.378952	-0.0562	-0.34227	0.494669	-0.33997	0.101576	0.100977	-0.49011	0.05467	0.077701	0.103037
POU2AF1	0.705275	-0.21882	0.272095	0.124857	0.340552	-0.70755	0.675929	0.08691	-0.14668	-0.43937	0.293426	-0.30244	0.021777	0.088859	-0.28707	-0.04944	-0.1816	0.007902
FAM46C	0.708553	-0.20219	0.451755	0.299329	-0.02894	-0.71936	0.50188	0.291585	0.056228	-0.40516	0.433401	-0.23001	0.055993	0.068591	-0.27065	0.106967	0.102099	-0.09406
IGJ	0.710303	-0.29629	0.451329	0.168245	0.012837	-0.74392	0.503509	0.125613	-0.11711	-0.24872	0.511258	-0.05347	0.031028	0.053078	-0.33465	0.003365	-0.02358	0.019165
IGHV3-15	0.71458	-0.25391	0.234927	0.24196	0.169477	-0.70663	0.710649	0.16602	-0.05474	-0.4018	0.447146	-0.41779	0.042921	0.10862	-0.29072	-0.11649	0.119483	-0.04652
IGHV3-11	0.723457	-0.38439	0.366188	0.281926	0.129358	-0.66992	0.534182	0.26826	-0.19996	-0.29237	0.389618	-0.30709	0.170169	0.010992	-0.47333	0.112895	0.07859	0.136456
LOC100132612	0.734192	-0.33522	0.569853	0.233103	-0.13079	-0.65648	0.283474	0.246368	-0.27236	-0.20446	0.541546	-0.17768	0.240031	0.147972	-0.44548	0.307186	-0.18503	-0.00707

IGHG1	0.735944	-0.29688	0.415966	0.338178	-0.0204	-0.68684	0.496806	0.311565	-0.17832	-0.26017	0.450871	-0.22241	0.10249	0.063222	-0.33385	0.18936	0.042319	-0.07309
FCRL5	0.737628	-0.27913	0.293947	0.098538	0.291073	-0.68353	0.611584	0.056743	-0.16966	-0.44119	0.336397	-0.43507	0.049857	0.134989	-0.28887	-0.13102	-0.11051	0.188074
IGLV1-44	0.745701	-0.33291	0.396544	0.546586	-0.00961	-0.66993	0.369825	0.55509	-0.35935	-0.04098	0.376399	-0.27735	0.330282	0.010801	-0.43773	0.294288	-0.03224	-0.01384
IGHM	0.746988	-0.27978	0.370117	0.440676	0.072992	-0.78267	0.490132	0.417422	-0.14527	-0.25504	0.353888	-0.12448	0.178586	-0.05089	-0.35287	0.063057	0.006417	0.147511
IGHV1-2	0.747806	-0.36162	0.309359	0.426247	0.050521	-0.72252	0.529523	0.39079	-0.16445	-0.23029	0.45403	-0.28951	0.240195	0.031557	-0.47444	0.036089	0.074016	0.070791
IGKV4-1	0.748572	-0.41582	0.416522	0.481655	-0.03993	-0.70972	0.480544	0.479206	-0.1146	-0.20627	0.41057	-0.12512	0.064674	-0.00851	-0.44196	0.148543	0.158499	0.028815
IGHV1-69	0.776135	-0.36163	0.450659	0.515432	-0.03993	-0.72778	0.415604	0.52142	-0.24739	-0.14381	0.460186	-0.23555	0.288791	0.031814	-0.48297	0.227111	-0.03778	0.036968
IGLC3	0.776771	-0.45121	0.418778	0.450947	-0.10405	-0.60162	0.377949	0.438445	-0.3187	-0.00989	0.407183	-0.14331	0.082927	0.033412	-0.41676	0.276684	0.076554	-0.07222
IGHV3-23	0.792846	-0.42504	0.3359	0.428887	-0.06101	-0.62008	0.53593	0.399684	-0.13252	-0.14446	0.481861	-0.38148	0.100055	0.078523	-0.42644	0.068461	0.27131	-0.11738
IGHV1-18	0.811332	-0.42633	0.341549	0.416161	0.041968	-0.65958	0.55987	0.409077	-0.17014	-0.23022	0.428515	-0.35784	0.153291	0.057978	-0.47839	0.075323	0.119967	-0.0176
IGKV3-20	0.814043	-0.27909	0.3674	0.3285	0.077181	-0.683	0.58246	0.301104	-0.17379	-0.25238	0.377467	-0.26477	0.124789	-0.02039	-0.29672	0.078527	0.049639	-0.00427
IGHV4-39	0.816083	-0.40148	0.31863	0.178576	0.100274	-0.63732	0.669568	0.133774	-0.05249	-0.29806	0.419505	-0.24018	-0.07269	0.036522	-0.36396	-0.09179	0.16125	-0.04163

Note: DEGs, differentially expressed genes.
