# Supplementary material for: Upregulated Expression of IL2RB Causes Disorder of Immune Microenvironment in Patients with Kawasaki Disease
Source: Biomed Res Int. 2022 Jul 25;2022:2114699. doi: 10.1155/2022/2114699 (PMC9343205; doi:10.1155/2022/2114699)
Supplement: Supplementary Materials — Supplementary Table 1: clinical data on children whose coronary artery tissues were tested in this study. Supplementary Table 2: the DEGs1 from the comparison between the untreated case group and the control group. Supplementary Table 3: the DEGs2 from the comparison between the treated case group and the control group. Supplementary Table 4: immune cell score matrix estimated by CIBERSORT algorithm. Supplementary Table 5: coexpression analysis of DEGs and immune cell populations. Supplementary Table 6: correlation analysis between the screened 15 core genes and CD4+ memory T cells. [file 2114699.f1.zip › Supplementary Table 6 (1).pdf]

S Table 6. Correlation analysis between the screened 15 core genes and CD4+ memory T cells.

	Resting CD4+ memory T cells		Activated CD4+ memory T cells	
	Coefficient	*P* value	Coefficient	*P* value
TRBV29.1	-0.11633501	0.453003807	0.778857622	**0.000798139**[b]
**TRAC**[a]	-0.510714286	**0.010578472**[b]	0.618771485	**0.00955495**[b]
**PLB1**[a]	-0.789285714	**0.000620867**[b]	0.609604501	**0.01760024**[b]
**IL2RB**[a]	-0.678571429	**0.002747759**[b]	0.582103546	**0.028082962**[b]
**IGHV5.51**[a]	-0.521930613	**0.018298911**[b]	0.515985364	**0.039224543**[b]
S1PR2	0.375	0.091493399	-0.490433696	0.074935378
TRAV21	-0.149077248	0.377824578	0.424011906	0.136853913
IGLV3.21	-0.558816169	**0.013193917**[b]	0.336100559	0.203826896
CCDC11P1	-0.492706185	0.060640234	-0.294868085	0.304766491
FILIP1L	-0.739285714	**0.02976657**[b]	0.238341609	0.249220774
ABCB6	0.371428571	**0.048754889**[b]	0.165005729	0.675624774
RNU1.19P	0.553175602	**0.042221214**[b]	-0.159561565	0.659928425
HMGB1P19	0.418415534	0.102610877	0.155077636	0.847767646
DCDC2C	-0.367414765	0.077788888	-0.101560736	0.823046796
RALYL	-0.424622787	0.091857047	-0.052255623	0.624809713

Note: [a]These genes are significantly correlated with these two types of immune cells ($P < 0.05$). [b]Significant *P* values less than 0.05 were all highlighted in bold.
